# Effective and Selective Ru(II)-Arene Complexes Containing 4,4′-Substituted 2,2′ Bipyridine Ligands Targeting Human Urinary Bladder Cancer Cells

**DOI:** 10.3390/ijms241511896

**Published:** 2023-07-25

**Authors:** Mathiyan Muralisankar, Jun-Ru Chen, Jebiti Haribabu, Shyue-Chu Ke

**Affiliations:** 1 Physics Department, National Dong Hwa University, Hualien 97401, Taiwan; murali6289@gmail.com (M.M.); 410114237@gms.ndhu.edu.tw (J.-R.C.); 2 Facultad de Medicina, Universidad de Atacama, Copayapu 485, Copiapo 1531772, Chile; haribabusiri970@gmail.com

**Keywords:** organoruthenium, ruthenium-arene complex, anticancer agent, bladder cancer

## Abstract

Cisplatin-based chemotherapy is a common regimen for bladder cancer, a life-threatening cancer with more than 500,000 new cases worldwide annually. Like many other metallodrugs, cisplatin causes severe side effects for its general toxicity. Organoruthenium is known for its structural stability, good anticancer activity, and possible low general toxicity. Here, we have prepared and characterized a series of water-soluble ruthenium-arene complexes with N,N′-chelating ligands: [Ru(II)-η^6^-arene-(4,4′-(X)_2_-2,2′-bipyridine)Cl]Cl (arene = *p*-cymene, X = C_4_H_9_ (**1**), COOH (**2**), COOCH_3_ (**3**), COOC_2_H_5_ (**4**); arene = benzene, X = C_4_H_9_ (**5**), COOCH_3_ (**6**), COOC_2_H_5_ (**7**)). These complexes are carefully characterized using single-crystal X-ray diffraction, UV-vis, IR, ^1^H NMR, and MALDI-TOF MS spectroscopy. Their DFT-calculated structural and thermodynamic properties are consistent with the experimental observations. Biophysicochemical studies of complex interaction with CTDNA and BSA supported by molecular docking simulations reveal suitable properties of **1**–**7** as anticancer agents. Cytotoxicities of **1**–**7** are evaluated on healthy human MCF-10a-breast epithelial and African green monkey Vero cells, and carcinoma human HepG-2-hepatic, T24-bladder, and EAhy-926-endothelial cells. All complexes exhibit much higher cytotoxicity for T24 than cisplatin. Particularly, **1** and **2** are also highly selective toward T24. Fluorescence imaging and flow cytometry demonstrate that **1** and **2** penetrate T24 cell membrane and induce early apoptosis at their respective IC_50_ concentrations, which ultimately lead to cell death. Statistical analysis suggests that the order of importance for T24 cell antiproliferation is protein binding, Log *p*, Ru–Cl bond length, while DNA binding is the least important. This study is the first to report the anti-bladder cancer efficacy of Ru-arene-2,2′-bipyridine complexes, and may provide insights for rational design of organoruthenium drugs in the enduring search for new chemotherapeutic agents.

## 1. Introduction

Cancer is a disease that causes abnormal cells of a body to proliferate uncontrollably and spread to surrounding healthy parts of the body, and is regrettably claiming the lives of millions annually [1]. The most common treatments for cancer are surgery, radiotherapy, and chemotherapy. Chemotherapy is a systemic treatment and is often used if the cancer has already spread or might spread in the future. Apoptosis has been considered a major mechanism of chemotherapy-induced cell death. Cisplatin as an anticancer agent has a venerable history in chemotherapy [2,3]. It kills cancer cells mainly by binding to nuclear DNA and subsequently interfering with the normal transcription and/or inhibiting DNA synthesis leading to apoptosis [4]. However, unlike target-specific surgery or radiotherapy, cisplatin in common with many other drugs can also affect some proliferating healthy cells and cause severe side effects, as they circulate throughout the body in the bloodstream. Also, cisplatin lacks activity or acquires drug resistance in several cancer cells. The balance of cytotoxicity and selectivity against cancer cells is an important indicator for evaluating new drugs. Drugs targeting specific proteins that control how cancer cells grow, divide, and spread have also been developed; however, they are undesirably prone to developing resistance over time. The advent of immune checkpoint inhibitors, which activate immune cells to recognize and destroy cancer cells, has revolutionized the way cancer is treated. Immunotherapy has the advantages of low toxicity and better treatment tolerance, and can also be used in combination with chemotherapy or targeted drugs to improve efficacy [5,6].

In the enduring search for new chemotherapeutic agents, Ru-complexes have emerged as potential candidates to replace cisplatin owing to their similar ligand exchange kinetics to those of cisplatin [7], reduced side effects in part by binding to serum transferrin and albumin, ability to adopt at least two stable oxidation states, and possibly novel mechanism of action. Both Ru(III) NAMI-A and KP1019 reached phase I clinical trials [8]. The higher solubility Na^+^ analogue, KP1339, of KP1019 (renamed as NKP-1339 or IT139) has entered phase II clinical trials. Both drugs are prodrugs and are activated by reduction before attacking the target cells. Both drugs were also proposed to act in a multifactorial and multitarget fashion, i.e., targeting both DNA and proteins, whereas the cytotoxicity of cisplatin is mainly associated with DNA binding.

**Figure 1:**
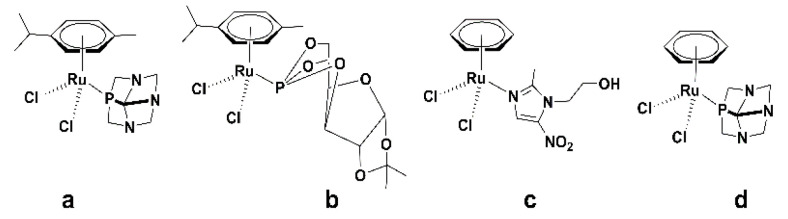
Chemical structures of (**a**) [Ru(η**^6^**-p-cymene)Cl_2_(pta)]; pta = 1,3,5-triaza-7-phosphaadamantane, (**b**) [Ru(η**^6^**-p-cymene)Cl_2_L] (L = sugar phosphite analogue), (**c**) [Ru(η**^6^**-benzene)Cl_2_ (metronidazole)], and (**d**) [Ru^II^(η^6^-benzene)Cl_2_(pta)].

Recently, the half-sandwich Ru(II)-arene organometallics have been shown to exhibit promising anticancer activity in vitro and in vivo [8]. These include the first reported drug candidate [Ru^II^(ɳ^6^-benzene)(Cl)(NH_3_)_2_]PF_6_ [9] and other examples [10,11,12,13] whose structures are shown in Figure 1. Their cationic forms have been assayed and shown good preclinical results. This class of complexes share a general formula [Ru^II^(ɳ^n+1^-arene)(X)(Y)(Z)]^n+^ (XYZ = monodentate) or [Ru^II^(ɳ^n+1^-arene)(X)(YZ)]^n+^ (X = monodentate, YZ=bidentate) and are characterized by a typical piano-stool geometry, wherein Ru is at the center, arene forms the seat of the stool, and X, Y, and Z make the three legs. They also share some other characteristics that give researchers momentum to search for new drugs in this class of complexes: (1) they are highly stable; (2) the π-bonded arene stabilizes the oxidation state of Ru(II), and exerts a kinetic trans effect to dissociate the chloride to activate the complex; (3) generally soluble, and its cationic counterpart is even more soluble in biological media; (4) the hydrophobic nature of the aromatic moiety improves permeability through cell membranes; (5) the chemical framework offers several ways to fine-tune the structure of arene or chelating ligands for more pronounced lipophilicity or nucleobase selectivity. All of these could lead to enhanced cytotoxic efficacy.

2,2′-bipyridine is a vital constituent in medicinal chemistry due to its coordination flexibility and biological versatility [14,15]. Surprisingly, the anticancer activity of the complexation of Ru(II)-arene and 2,2′-bipyridine derivatives has rarely been reported. To our knowledge, only Ru(II)-arene (arene = ɳ^5^-methyl-cyclopentadienyl) with 2,2′-bipyridine derivatives for higher anticancer activity in ovarian cancer cells was reported [16]. On the other hand, 2,2′-bipyridine in place of the chelating ethylenediamine for [RuCl(η^6^-arene)(ethylenediamine)]^+^ rendered the subsequent complex nearly inactive against ovarian and lung cancer cells. However, the replacement of ethylenediamine with acetylacetone or bipyridyl diol resulted in enhanced cytotoxicity. Equally surprising, there have not been any reports of Ru-arene complex against bladder cancer, a common malignant tumor in the human urogenital system, for which many patients are inherently or become refractory to the conventional cisplatin-based chemotherapy and are waiting for new chemotherapeutic drugs to target and kill the bladder cancer cells. To date, the assay of Ru-arene complex against bladder cancer cells has not been reported. In this context, we report a series of novel half-sandwich Ru(II)-arene (arene = ɳ^6^-p-cymene or ɳ^6^-benzene) with 2,2′-bipyridine-4,4′-para-substituted (tert-butyl, carboxylic acid, and ester) as chelating ligands that target human urinary bladder cancer T24 cells with much higher antitumor activity and selectivity than cisplatin.

## 2. Results

### 2.1. Synthesis of Complexes

**Scheme 1:**
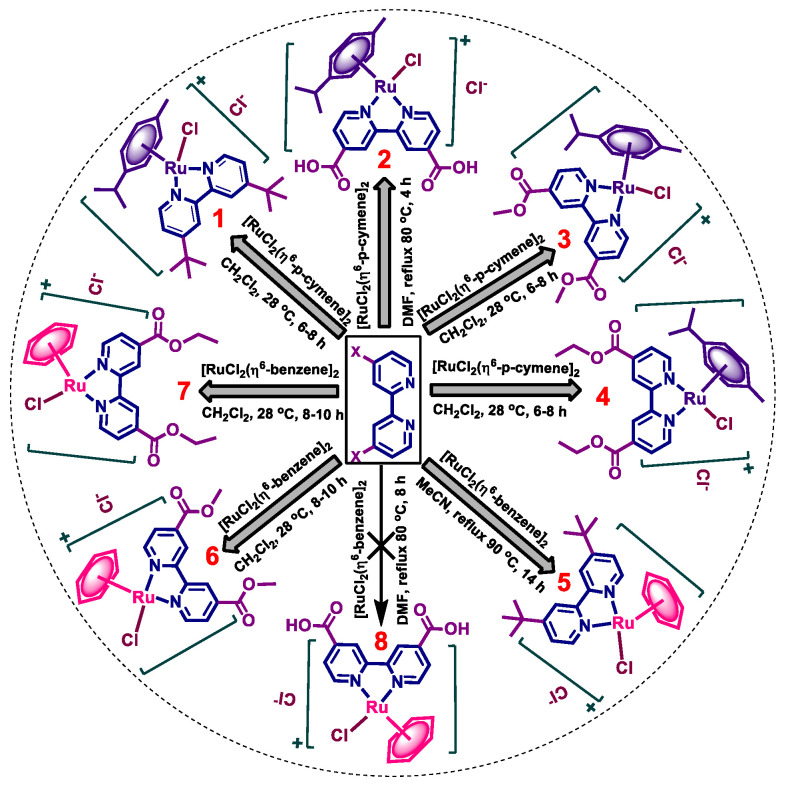
Synthetic route for the preparation of complexes **1**–**7**.

The synthetic routes of [Ru-η^6^-arene-(4,4′-(X)_2_bipy)Cl]Cl (arene = p-cymene, bipy = 2,2′-bipyridine, X = C_4_H_9_ (**1**), COOH (**2**), COOCH_3_ (**3**), COOC_2_H_5_ (**4**); arene = benzene, X = C_4_H_9_ (**5**), COOCH_3_ (**6**), COOC_2_H_5_ (**7**)) are summarized in Scheme 1. Complexes **1**–**7** are obtained as air-stable orange solids. Complexes **1** and **3**–**7** are readily soluble in water, whereas **2** is more soluble in high polar organic solvents.

### 2.2. Spectroscopic Characterizations

#### 2.2.1. UV-Visible Spectroscopy and Stability Experiments

**Figure 2:**
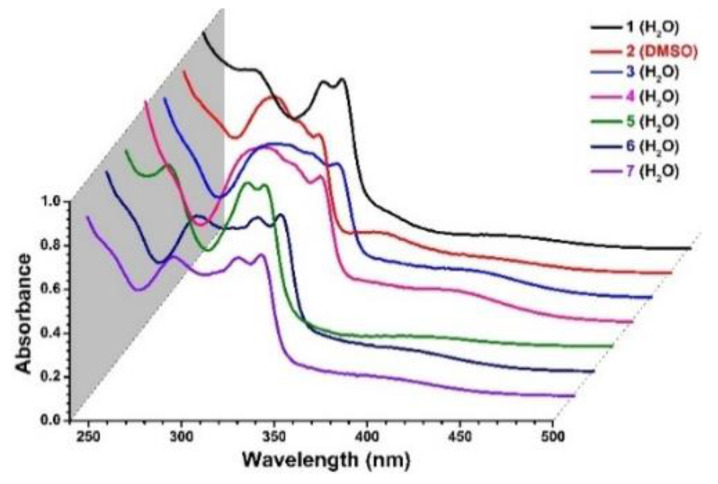
UV-Vis spectra of **1**−**7** at 15 × 10^−6^ M.

UV-visible spectra of **1**–**7** are shown in Figure 2. The absorption bands around 247–264 and 285–320 nm, observed as a continuous broad band, are assigned to the ligand-centered π→π* and n→π* transitions, respectively. In addition, all complexes show similar broad absorption bands centered at ~410 nm. This band is ascribed to metal-to-ligand transition wherein charge transfer takes place from the occupied HOMO of Ru(II) to the LUMO π*(–C=N–) of the 2,2′-bipyridine ligand. 

Complexes **1**–**7** are also monitored for their aqueous stability in PH 7.2 Tris-buffered solution at NaCl concentration of 4 mM and 110 mM, using UV-Vis spectroscopy (Figure S1). Neither additional absorption peaks nor optical density changes were observed over a period of 20 h, confirming their stability under the respective physiological-like conditions.

#### 2.2.2. FT-IR Spectroscopy

**Figure 3:**
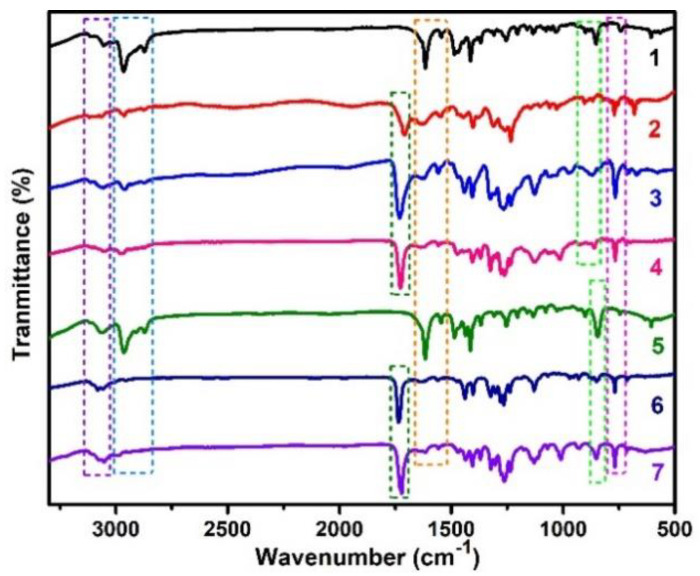
FT-IR spectra of **1**–**7**. Dotted box mark from left to right: purple, ʋ (C-H); blue, ʋ (–CH_3_); dark green, ʋ (C=O); orange, ʋ (C=C) and ʋ (C=N); light green, arene ʋ (C-H) out of plane bending; purple, 2,2′-bipyridine ʋ (C-H) out of plane bending.

Figure 3 shows the FT-IR spectra of **1**−**7** in support of the variation in functional groups. The aromatic ʋ(C-H) stretchings are observed in between 3130 and 3030 cm^−1^. The ʋ(–CH_3_) stretchings from the p-cymene and/or the 2,2′-bipyridine rings are observed in the 3020–2850 cm^−1^ range. The ʋ(C=O) stretching for the carboxyl of the 2,2′-bipyridine rings is observed at 1732–1711 cm^−1^ for all complexes except **1** and **5** wherein carboxyl group is absent. The ʋ(C=C) and ʋ(C=N) stretchings of the 2,2′-bipyridine rings are observed at 1616 and 1554 cm^−1^, respectively, for all complexes. The symmetric ʋ(C-H) out of plane bending for η^6^-p-cymene for **1**–**4** is observed at ~888 cm^−1^ whereas that for η^6^-benzene is observed at ~843 cm^−1^ for **5**–**7**. The symmetric ʋ(C-H) out of plane bending for 2,2′-bipyridine rings is observed at ~770 cm^−1^ for all complexes. The FT-IR data confirm the formation of the respective complexes.

#### 2.2.3. NMR Spectroscopy

**Figure 4:**
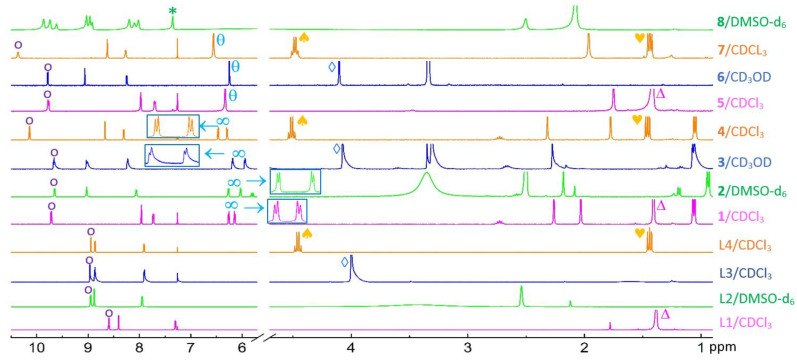
Coordination of 4,4′-substituted 2,2′ bipyridine ligands to Ru(II)-arene complexes: ^1^H-NMR of the aromatic (10.5–7.0 ppm), the arene (7.0–5.8 ppm), and the aliphatic region (5.65–1.0 ppm) followed through each individual compound preparations (see the text for details). Full spectra for the ligands and complexes are given in Figures S2 and S3.

The coordination of 4,4′-substituted 2,2′-bipyridine ligands to Ru(II)-arene complexes are monitored using 400 MHz ^1^H NMR (Figure 4). The two aromatic protons adjacent to the nitrogens of 2,2′-bipyridine (L1-L4) labeled “o” are observed at 8.96–8.59 ppm, whereas the same protons in **1**–**7** appear at 10.36–9.64 ppm. The deshielding effect evidences the coordination of 2,2′-bipyridine to Ru(II) forming Ru-N bonds. The presence of tert-butyl groups of L1, **1** and **5** is observed “Δ”at ∼1.40 ppm. No peak for the carboxylic acid groups of L2 and **2**, expected at ~11 ppm, was detected. However, the ʋ(C=O) stretching observed by FT-IR confirms the presence of carboxyl group of **2** (Figure 3). The methoxy groups in L3, **3**, and **6** are observed “◊” at ~4.10 ppm. The ethyl groups of L4, **4**, and **7** produce a quartet “♠” at ~4.5 ppm and a triplet “♥” at ~1.45 ppm. The p-cymene moiety of **1**–**4** are characterized by the two doublets “∞” in between 6.47 and 5.94 ppm (see inserts, Figure 4), whereas the singlet peak “θ” at 6.34, 6.24, and 6.55 ppm confirm the presence of benzene of **5**, **6**, and **7**, respectively. The targeted complex **8** was not obtained under standard reaction condition; the benzene group was displaced from the metal center during the reaction and characterized by the free benzene sharp singlet “∗” at 7.34 ppm.

#### 2.2.4. MALDI MS Spectroscopy

**Figure 5:**
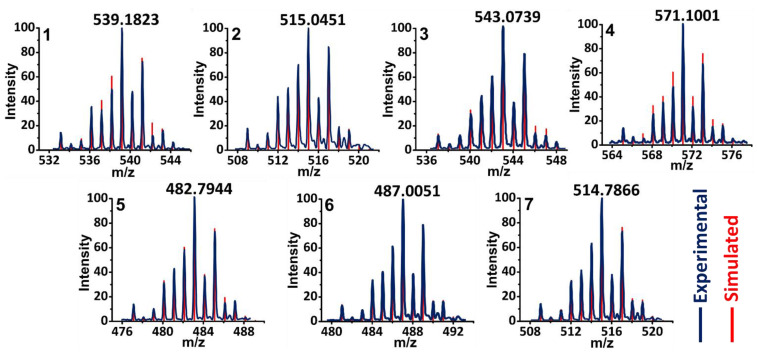
MALDI-TOF mass spectrometry: experimental data (blue dotted peaks) and isotopic pattern simulation (red sticks). Simulated: [**1-Cl**]^+^: C_28_H_38_ClN_2_Ru (539.17615); [**2-Cl**]^+^: C_22_H_22_ClN_2_O_4_Ru (515.0306); [**3-Cl**]^+^: C_24_H_26_ClN_2_O_4_Ru (543.0619); [**4-Cl**]^+^: C_26_H_30_ClN_2_O_4_Ru (571.0932); [**5-Cl**]^+^: C_24_H_30_ClN_2_Ru (483.1136); [**6-Cl**]^+^: C_20_H_18_ClN_2_O_4_Ru (486.9993); and [**7-Cl**]^+^: C_22_H_22_ClN_2_O_4_Ru(515.0306).

MALDI-TOF spectra of this series of neutral [RuCl(η^6^-arene)(bipy)(X)_2_]Cl (**1**–**7**) were collected in positive-ion mode and detected as singly charged cations (Figure 5). The cluster of peaks exhibits an isotopic pattern from one ruthenium, and one chlorine as well. Simulated isotopic patterns for the corresponding cations of **1**–**7** generated via the dissociation of the chloride counterion exactly matched the experimental mass spectra. Combined with the complex stability studies in Section 2.2.1, it is shown that the Ru–Cl bond may not be inherently labile in this series of complexes.

### 2.3. Structure Analysis

**Figure 6:**
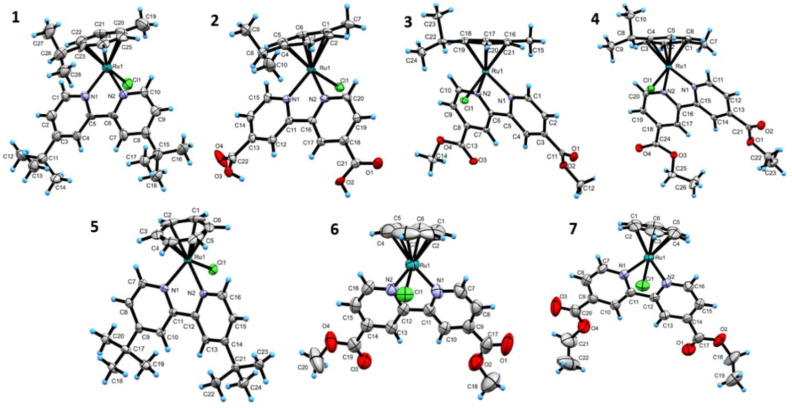
ORTEP drawing of **1**–**7** with thermal ellipsoids scaled at different probabilities, and with the chloride counterion removed for clarity.

The orange color crystals for X-ray crystal structure analysis are obtained by slow evaporation of dichloromethane or hexane solutions of the complexes. Thermal ellipsoid plots of **1**–**7**, along with the crystallographic numbering schemes, are shown in Figure 6. The central Ru(II) is coordinated by the six carbon atoms of the arene ring, the N(1), N(2) of the bidentate 2,2′-bipyridine ligands, and one terminal chlorido to give a pseudo-octahedral geometry about the metal center. The shape of the complexes resembles a half-sandwich “piano-stool” geometry, wherein the π-bonded η^6^-C_6_H_6_ serves as the seat and the Ru-N(1), Ru-N(2), and Ru-Cl1 serve as the three legs [17]. The Ru to the ɳ^6^-carbons have similar bond distances. The 2,2′-bipyridine is arranged in a *cis*-conformation about the C–C bond of 2,2′-bipyridine. The sp^2^-hybridized pyridyl *N*-donors coordinate to the Ru and form a five-membered chelate ring, which is commonly found in 2,2′-bipyridine complexes [18]. The average Ru–N and N1–N2 bond distances are 2.09 Å and 2.59 Å, respectively. The average N1–Ru–Cl1, N2–Ru–Cl1, and N1–Ru–N2 bond angles are 85.6°, 84.3°, and 76.8°, respectively. Selected crystallographic data are given in Tables S1 and S2. The crystallographical parameters are similar to the reported values for comparable complexes [19,20].

### 2.4. DFT Studies of Structure and Log p

DFT computations at B3LYP, M06-L, and ωB97XD levels of theory are performed to study the structural and thermodynamic properties of **1**–**7**. The optimized structures differ slightly between the different functionals; however, they all agree with corresponding crystallographic structures to within ~±0.05 Å and ±2° precision. The absence of imaginary vibrational frequency for each optimized structure ensures that they correspond to equilibrium stationary minima.

**Figure 7:**
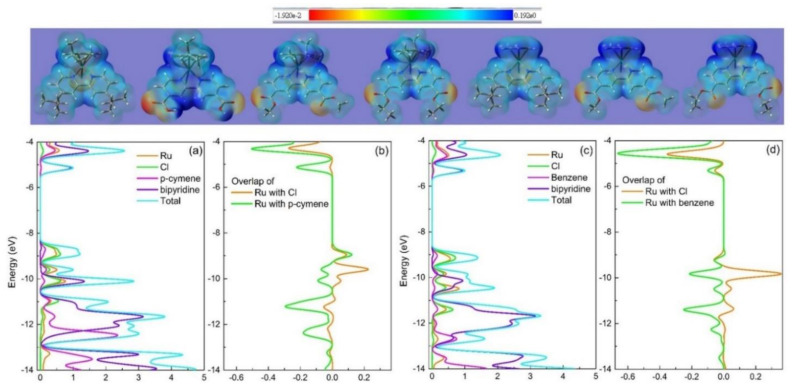
Top: ESP-mapped electron density surface (isovalue = 0.004 au) of **1**–**7** (left to right). The red color represents the most negative regions of the ESP (−0.0192 au), and the deep blue color indicates the least electron density of the ESP (0.192 au). Bottom: (**a**) PDOS of **1**, (**b**) COOP diagram corresponding to the PDOS of **1**, (**c**) PDOS of **5**, (**d**) COOP diagram corresponding to the PDOS of **5**. DFT method: B3LYP with SDD on Ru and 6-31+g(d,p) for other atoms in gas phase.

We are particularly interested in the Ru–Cl bond length (Table 1), as elongated Ru–Cl bond length may lead to higher chemical reactivity by facilitating the departure of chloride ion required for drug activation [14]. Complex **1** exhibits the longest Ru–Cl bond length. As will be shown later, **1** indeed exhibits the highest cytotoxicity against T24 cancer cells among all seven complexes. Figure 7 (top) maps the electrostatic potential (ESP) of **1**–**7** onto their respective electron density surfaces, which describe the shape and size of the complexes. The ESP energy value at a point on the surface is color-coded, which describes the richness or poorness of electron density. Complex **1** exhibits the highest electron density around Ru(II) as indicated by its lightest blue color, while **5**–**7** exhibit relatively deeper blue around Ru(II) implying lower electron densities. This observation is qualitatively consistent with changes of Ru–Cl bond length. Complex **1** differs from the structure of **5** only in the para-substitutions of benzene ring with a methyl and an isopropyl, but do lengthen the Ru–Cl bond length by 0.02 Å (Table 1, XRD). Thus, **1** and **5** form an ideal pair that serves as an example for extracting the percent contribution of each moiety to the molecular orbital and its bonding characteristics that may quantitatively highlight the potential influence of the arene group on the Ru–Cl bond length.

The projected density of states (PDOS) spectra [21] of **1** and **5** (Figure 7a,c) show that the highest occupied molecular orbital (HOMO) and HOMO-1 are largely Ru–Cl-based (HOMO: 37% Ru, 45% Cl for **1** at −8.68 eV; 32% Ru, 50% Cl for **5** at −9.02 eV; HOMO-1: 44% Ru, 55% Cl for **1** at −8.95 eV; 46% Ru, 53% Cl for **5** at −9.23eV). The bipyridine ligands contribute little to the HOMO and HOMO-1, whereas the lowest unoccupied molecular orbital (LUMO) is bipyridine dominated (94% for **1** at −5.13 eV, 92% for **5** at −5.31 eV). The peaks for the Ru-Cl in the frontier region of the occupied molecular orbitals locate at 0.34 eV (HOMO) and 0.28 eV (HOMO-1) higher in energy for **1** than for **5**. This evidences greater electron donation from p-cymene of **1** to the Ru center as compared to benzene of **5**, in support of the ESP-mapped electron density surface plot (Figure 7 top). This is also evidenced by the crystal orbital overlap population (COOP) plot (Figure 7b), as a complement to the PDOS plot, which partitions the overlaps into bonding and antibonding regions and shows a bonding interaction between Ru and p-cymene with respect to the HOMO-1 orbital. In addition, the COOP curves (Figure 7b,d) show that Ru–Cl bonds corresponding to the HOMO-2 orbital are bonding interactions and the bonding strength of **5** is 1.636 times stronger than that of **1**, thus is responsible for the shorter Ru–Cl bond length of **5**. Apparently, para-substitutions of benzene ring with a methyl and an isopropyl have measurable impacts on the electron density distribution around Ru(II) and is responsible for the lengthening of the Ru–Cl bond.

The Ru–Cl bond length is by no means the only factor responsible for the excellent cytotoxicity of **1** against T24 cancer cells; other factors, such as a key metric of a drug molecule, Log *p*, should also play an important role. Log *p*, the logarithm of the water-octanol partition coefficient, provides a measure of permeability of a complex through a lipid environment. The higher the Log *p*, the more likely the complex is to penetrate the lipid bilayer of a cell membrane and act as a drug for intracellular targets. The experimental Log *p* values of **1**–**7** are measured and listed in Table 1. Complexes **1** and **5,** bearing mildly hydrophobic 4,4′-di-tert-butyl substituents, exhibit much higher Log *p* than other complexes. The p-cymene-bearing complexes are more lipophilic than their benzene-carrying counterparts.

The water-octanol Log *p* value of an organometallic complex can also be obtained by predictions [22,23,24]. For comparison with the Log *p* of **1**–**7** predicted by the empirical fragment-based Log *p* calculator miLogP of Molinspiration [25], we performed quantum mechanical calculations using B3LYP, M06-L, and ωB97XD functionals with the SMD implicit solvent model. Although miLogP and all three DFT functionals overestimate the Log *p*, they lie in orders reasonably parallel to the experimental trend for **1**–**7** (Table 1). The best performing method is ωB97XD, which yields the lowest mean signed deviation (MSD) of 1.96 Log *p* unit relative to the experiment, and is selected for further examination. The superiority of ωB97XD is attributed to its ability to capture both short- and long-range interactions, whereas conventional B3LYP or M06-L used in this study only capture short-range interaction. Since the Gibbs free energy used to calculate Log *p* depends on experimental conditions, the Log *p* prediction is improved by changing the dielectric constant 9.863 of n-octanol to 8.1 to account for the effect of water saturated n-octanol [26,27]. Correspondingly, the MSD is reduced to a satisfactory value of 0.46 Log *p* unit (Table 1). The Log *p* predictions could be further improved by considering other DFT functionals, improving the quality of the basis sets, or including MD conformers, but they are beyond the scope of this study [28].

### 2.5. Interaction of Ru(II)-Arene-4,4′-Para-Substituted-2,2′-Bipyridine with DNA

#### 2.5.1. Determination of Complex/CTDNA Binding Constants

**Figure 8:**
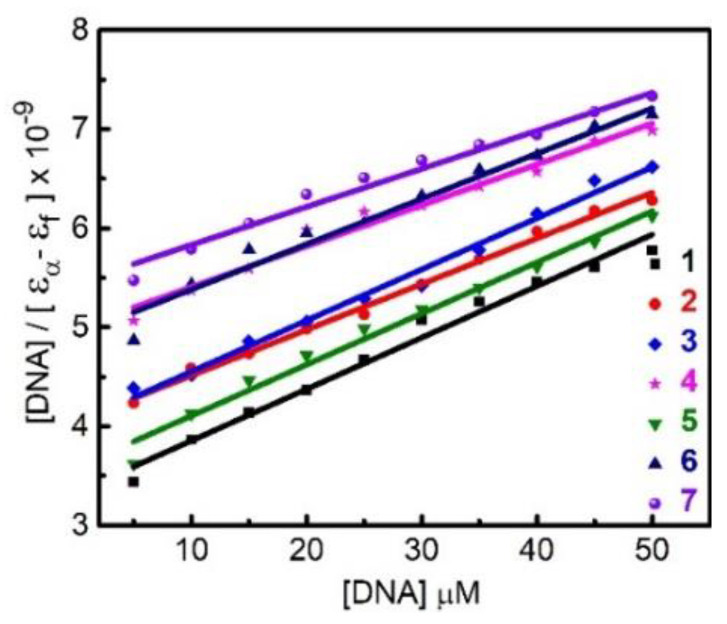
Plots of [DNA]/(*ε*_a_ − *ε*_f_) *versus* [DNA] for the titration of **1**–**7** with CT-DNA.

The ability of **1**–**7** to interact with calf thymus DNA (CTDNA) is assessed by titration of the complex kept at a constant concentration against the linearly increasing amount of CT-DNA and monitored spectrophotometrically. Figure S4 shows that continuous hypochromic effects on the π-π* transition of the 2,2′-bipyridine ligand centered at ~300 nm are observed during the course of titration. The hypochromism is indicative of non-covalent interaction between the aromatic chromophore of the complex and the base pairs of DNA. The intrinsic-binding constants (*K_b_*) are derived based on the [DNA]/(ε_a_ − ε_f_) versus [DNA] plot (Figure 8) and summarized in Table 2. The magnitude of *K_b_* follows the order of **7** > **6** > **4** > **3** > **2** > **5** > **1**.

#### 2.5.2. Determination of EB/CTDNA Fluorescence Quenching Constants

**Figure 9:**
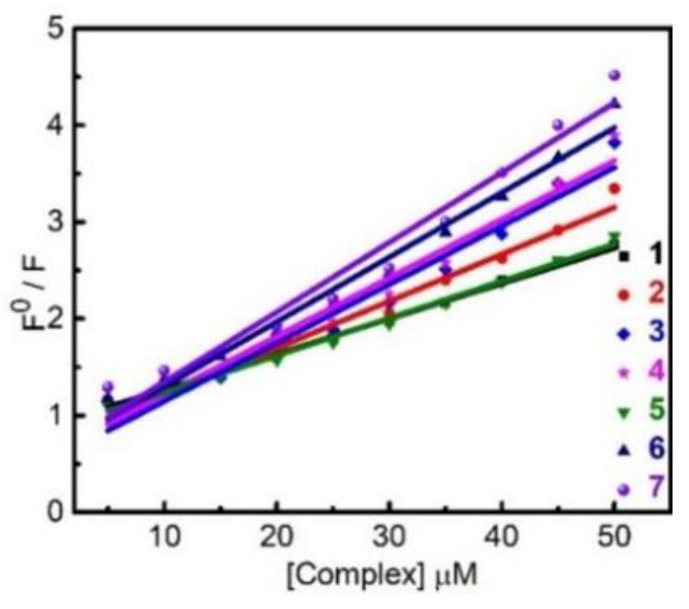
Stern–Volmer plots of fluorescence titrations of EB-CTDNA with **1**–**7**.

To learn more about the binding properties of **1**–**7** with CTDNA, the fluorescence competition technique is employed. EB is an orange fluorescent dye and an intercalating agent for DNA. When EB intercalates between adjacent DNA base pairs with its planar phenanthridine moiety to form EB-CT DNA adduct, the hydrophobic environment of DNA protects EB from the strong fluorescence quencher water and thus intensifies the fluorescence of EB by a factor of ~12 (Figure S5). Titration of EB-CTDNA adduct with nonfluorescent **1**–**7** gradually quenches the fluorescence of EB (Figure S5). This means that **1**–**7** are able to compete with greater binding affinity for DNA than that of the preloaded EB. Figure 9 shows the Stern–Volmer plots of the fluorescence titration experiments, from which the derived quenching constant (K_q_) and apparent DNA binding constant (K_app_) are summarized in Table 2 (see SI for details). The magnitudes of K_q_ and K_app_ of **1**–**7** follow the same order of **7** > **6** > **4** > **3** > **2** > **5** > **1** as the intrinsic binding constants *K*_b_. All seven complexes exhibit good binding affinity toward DNA when compared with literature values [29,30,31,32].

#### 2.5.3. Viscometric Measurements and DNA Fragmentation Assay

**Figure 10:**
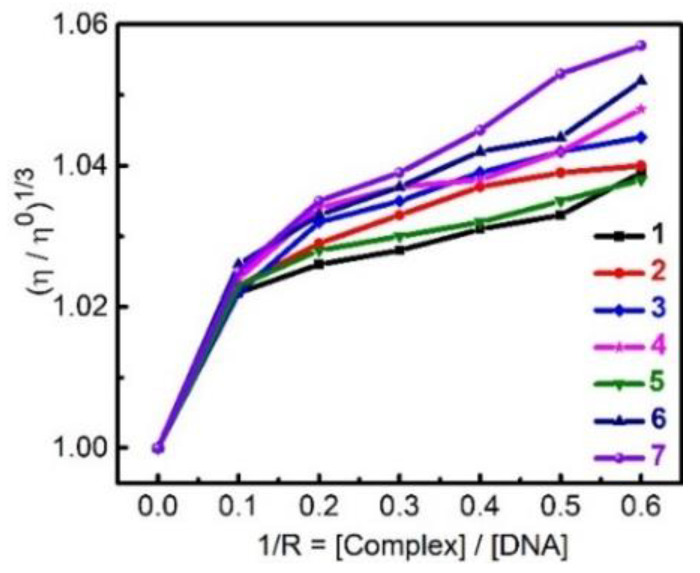
Effect of increasing amounts of **1**–**7** on the relative viscosity of CTDNA.

Viscometric measurement is sensitive to DNA helix length changes, and the greater the DNA helix length is, the higher the viscosity is. Figure 10 shows that the viscosity of CTDNA in water gradually increases with the addition of the binder **1**–**7**, and the effect of **1**–**7** binding on the relative viscosity of CTDNA follows the same trend observed for k_b_, k_q_, and k_app_. However, the increases of relative viscosity are very limited. The common value for EB as a classical intercalator is about 1.5, whereas for **7** it is 1.057, at [EB]/[CTDNA] = 0.6. This phenomenon suggests that the mode of **1**–**7**/CTDNA interaction is groove binding rather than the classical intercalating. The DNA fragmentation assays show that **1**–**7** do not cleave DNA (Figure S6).

#### 2.5.4. Molecular Docking with DNA

**Figure 11:**
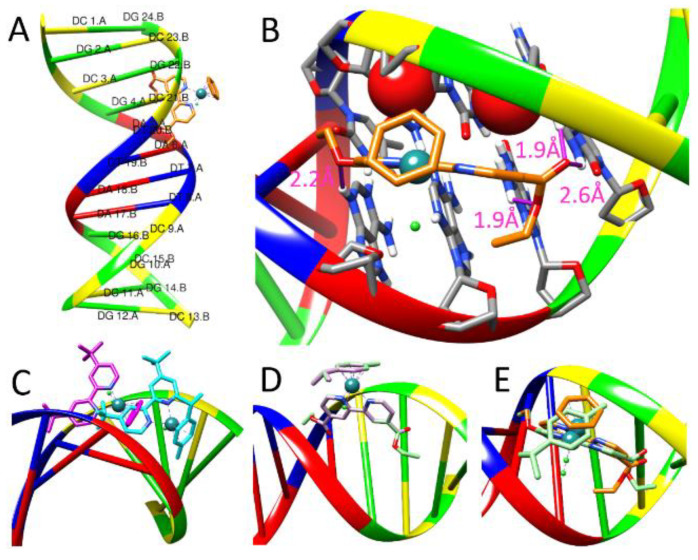
(**A**) Overall view of the best docked pose of **7** in dodecamer 1BNA. (**B**) Close-up view of (**A**) showing oxygen atoms, red sphere in VDW radius, of deoxyribose stacking over the bipyridine rings. H-bonds colored in pink stick. (**C**) Best docked poses of **1** (cyan) and **5** (pink) showing partial insertion of the bipyridine ring. Comparison of best docked poses of (**D**) **4** (green) and **3** (light purple), and (**E**) **7** (orange) and **4** (green). See text for details.

We conduct molecular docking studies to gain insight into the binding fashion of **1**–**7** to CTDNA, modeled with a B-DNA dodecamer (PDB ID: 1BNA) (Figure 11A). The calculated binding potency trend (free energy of binding, ΔG, Table 2) of best docked structures, **7** > **6** > **4** > **3** > **2** > **5** > **1**, fully agrees with the spectroscopic trend, implying that the mode of **1**–**7** interactions with CTDNA can be rationally described by their interactions with 1BNA.

All complexes are docked in the minor groove of 1BNA, which agrees with the viscosity measurements. Figure 11A,B show that the planar 2,2′-bipyridine moieties of **7** are tucked into the minor groove and form stacking interactions with the oxygen atoms of two adjacent deoxyriboses of the backbone. The Ru-arene moiety lies above the edge of the backbone. The terminal 4,4′-bis(ethoxycarbonyl) makes hydrogen bindings to the base pairs (as indicated by pink sticks in Figure 11B). The bulky 4,4′-di-tert-butyl of **1** and **5** prohibits full insertion of 2,2′-bipyridine into the groove (Figure 11C), and thus places **1** and **5** at the bottom two of the binding potency trend. Complex **4** overlaps with **3** (Figure 11D), but its extended alkyl makes additional van der Waals contact with the groove and results in a higher binding potency than **3**. Compared with **7**, the methyl group of ɳ^6^-p-cymene of **4** forces its 2,2′-bipyridine to tilt about 12^0^ from the backbone (Figure 11E), lessening the stacking interactions between the two pyridyl rings and the two oxygen atoms of deoxyribose and resulting in **4** having a lower binding affinity than **7**.

Overall, the docking analyses reveal that (1) the longer the substituent at the 4,4′-position of bipyridyl, the higher the affinity, and (2) the less bulky Ru-ɳ^6^-benzene complexes bind more strongly to the groove than the Ru-ɳ^6^-p-cymene complexes, which fully accounts for the spectroscopic trend.

### 2.6. Interaction of Complex ***1***–***7*** with Protein

#### 2.6.1. Fluorescence Titration Studies

**Figure 12:**
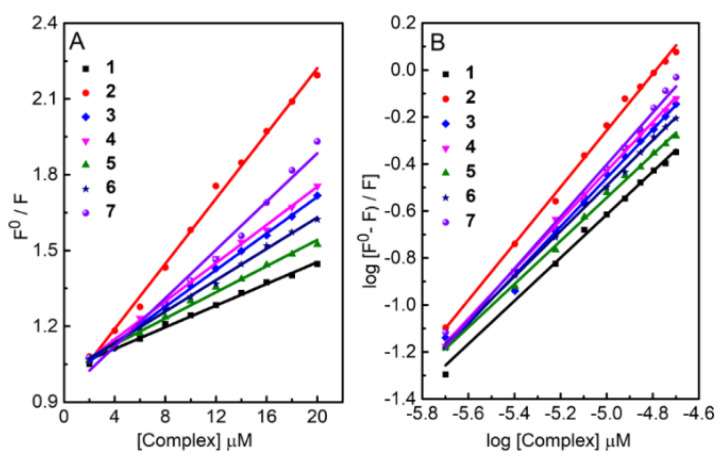
(**A**) Stern–Volmer (**B**) Scatchard plots of fluorescence titrations of **1**–**7** with BSA.

Bovine serum albumin (BSA) exhibits strong intrinsic tryptophan fluorescent emission at 345 nm when excited at 280 nm. To explore the mode of complex-protein interaction, we monitor the BSA fluorescence quenching at 345 nm by addition of increasing amounts of **1**–**7** to a fixed quantity of BSA (Figure S7). The reduced fluorescence demonstrates the formation of complex-protein adduct and perturbation to the local structure of tryptophan. The molar absorptivity of tyrosine and phenylalanine are 10- and 200-fold weaker than tryptophan, respectively, and are ignored in the modelling [33]. The observed hypochromicity offers evidence of hydrophobic interaction [34] between **1**–**7** and BSA. The complex binding-induced fluorescence quenching is described by the linear Stern–Volmer relationship (Figure 12A) to obtain the quenching constant *K*_q_ (see SI for details). Further, the equilibrium binding constant (*K_b_*) of the complex to BSA and the number of binding sites (n) are obtained by casting the fluorescence intensity in the absence of quencher (*F^0^*) and quencher concentration [Q] dependent *F* into the Scatchard equation [35]: log [(*F*^0^*− F)/F*] = log *K_b_* + *n* log[Q], as shown in Figure 12B. Table 3 summarizes the derived *K*_q_, *K_b_* and n for all complexes. The *K_b_* and *K_q_* follow the same order as **2** > **7** > **4** > **3** > **6** > **5** > **1**. Complexes **2** and **7** stand out as particularly attractive to BSA among all complexes tested. 

#### 2.6.2. MALDI-TOF MS Study

The binding of **2** and **7** to BSA are further confirmed by using MALDI-TOF MS (Figure S8), which shows that the mass of **2**- or **7**-bound BSA is higher than that of BSA alone. 

#### 2.6.3. Molecular Docking Study

**Figure 13:**
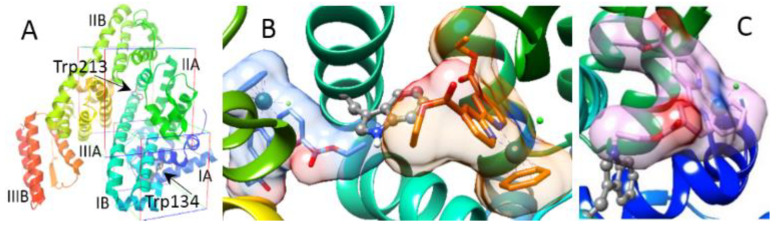
(**A**) BSA consists of I, II, and III homologous domains, each of which is divided into A and B subdomains (PDB ID: 4f5s). Molecular docking calculations center on Trp134 and Trp213 with a grid box of 18 Å × 18 Å × 18 Å and 22.5 Å × 22.5 Å × 22.5 Å, respectively. The grid size of each search space is large enough to accommodate the site of a flexible complex and its interacting residues. (**B**) Binding pose of **7** at the back (blue) and in front (orange) of Trp213 (ball and stick). (**C**) Binding pose of **7** in the Trp134 pocket.

To gain insights into the **1-7**/BSA interactions, we perform docking studies. BSA has two tryptophans (Figure 13A) [36]. Trp134 is located on the surface of sub-domain IA. Trp213 is located within a hydrophobic pocket in sub-domain IIA surrounded by sub-domains IB, IIB, and IIIA and is less accessible than Trp134. The Trp213 pocket has two binding regions, the front and back sides of the indole ring of Trp213, whereas Trp134 has only one binding region. Binding is considered as an active pose only if there exists at least one van der Waals contact between the complex and the Trp. A certain degree of binding specificity is observed. Complexes **5**–**7** bind to both Trp pockets, whereas **1**–**4**, carrying a bulky η^6^-p-cymene moiety, could bind to only the exposed Trp134 pocket, due to steric hindrances prohibiting their entrance to the Trp213 pocket located deeper into the BSA interior. Figure 13B and 13C show the orientation and molecular surface of the primary active binding poses of **7** in the Trp213 and Trp134 pocket, respectively. The interactions between **7** and residues surrounding the tryptophan are illustrated in Figure S9. Binding to either one of these regions perturbs the local conformation of the tryptophan pocket and leads to quenching of the intrinsic fluorescence as observed in Figure 12A,B.

Table 3 lists the lowest binding energy of active poses for **1**–**7** bound to Trp134 or Trp213 pockets. Complexes **2** and **7** have the lowest binding energies and thus are most potent as BSA binders, which agrees with the spectrofluorometric data showing **2** and **7** are the most effective fluorescence quenchers for BSA among the seven complexes. Based on these findings, the biochemical aspects involved in the BSA-Ru-arene interaction is also better understood. 

### 2.7. Anticancer Activity of Ru(II)-Arene Complexes

#### 2.7.1. MTT Cell Proliferation Assay

The cytotoxicities of these Ru(II)-arene-bipyridine complexes are evaluated on five cell lines, including healthy human MCF-10a-breast epithelial and African green monkey Vero cells, and carcinoma human HepG-2-hepatic, T24-bladder, and EAhy-926-endothelial cells, under identical conditions using the MTT assay at 24 h incubation (Table 4, see SI for details). The concentration-dependent in vitro cytotoxic potency plots are given in Supporting Figure S10. Table 4 compares the derived half-inhibitory concentration IC_50_ values for **1**–**7** against five cell lines with those for the widely used anticancer agent cisplatin. Complexes **1**–**7** (except **4**) are less cytotoxic to healthy MCF-10a and Vero cells than cisplatin, indicating that the general toxicity of this series of complexes is lower than that of cisplatin, and therefore more selective. This supports the collective hypothesis that is Ru-arene complexes usually exhibit lower general toxicity than cisplatin [37,38].

The most exciting finding is that most of the complexes in this work exhibit much higher cytotoxicity than cisplatin in T24 cancer cells. Among them, **1** exhibits the highest activity, being at least 4.2 times more potent than cisplatin. Complexes **1**–**4** carrying p-cymene are about twice as effective as their benzene-carrying counterparts. Remarkably, **2** is not only cytotoxically effective but also highly selective toward T24, as **2** is not toxic to other cell lines at concentrations below 50 μM. This suggests that **2** could be potentially selective toward T24 in vivo. Complex **1** is more cytotoxic than **2** in T24, but is slightly less selective than **2**. This finding constitutes the first report of the efficacy of Ru-arene with 4,4′-para-substituted-2,2′-bipyridine ligands against human bladder cancer, which is promising.

Serendipitously, **6** also effectively inhibits the growth of HepG-2 with an IC_50_ value of 14.5 μM compared to 49.1 μM for cisplatin. The IC_50_ values of **6** are much higher for the other three cell lines, indicating the likely selectivity of **6** for HepG-2 in vivo. 

#### 2.7.2. Detection of Cell Death by Acridine Orange/Ethidium Bromide Staining and Quantification by Flow Cytometry

**Figure 14:**
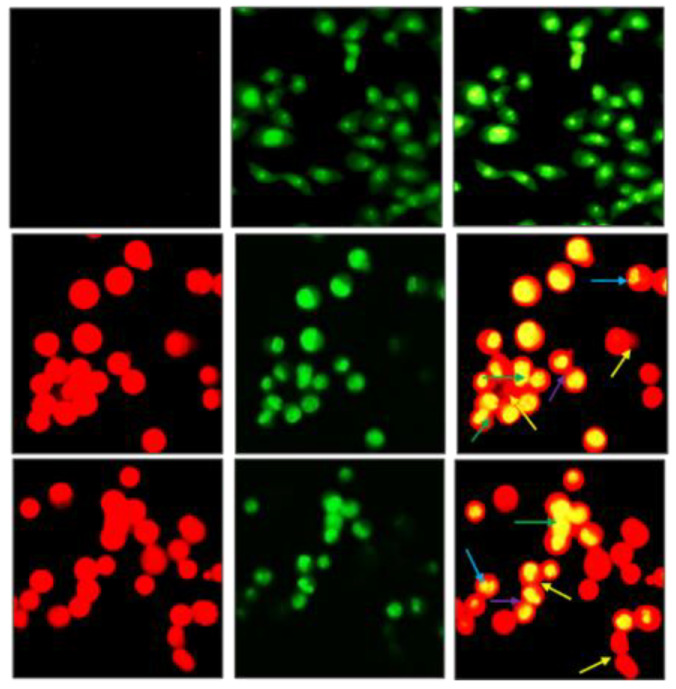
Fluorescence microscopy images of control (row 1) and T24 cells treated with **1** (row 2) and **2** (row 3) at IC50 concentration after 24 h incubation. Arrows: Cell and chromatin condensation (green), membrane blebbing and folding (yellow), nuclear fragmentation (blue) and formation of apoptotic bodies (purple).

Figure 14 provides a visualization of the morphological changes of T24 cells induced by treatment of 1 and 2 at IC50 concentration. The cells are stained with green acridine orange (AO), which is membrane-permeable for both viable and non-viable cells, and with red ethidium bromide (EB) which is permeable through damaged cell-membrane (late apoptotic or non-viable cells). The viability of cells is thus determined based on membrane integrity and the differential uptake of AO and EB. For the untreated cells (row 1), no red EB stain is observed, showing all cells are viable. The cells become non-viable after treatment with 1 and 2, as evidenced by the red staining from EB (rows 2 and 3). When the AO and EB images are merged, the cells exhibit a bright orangish yellow nucleus and a red cytoplasm with typical morphologic features of apoptosis including chromatin condensation, membrane blebbing, nuclear fragmentation, and formation of apoptotic cells. In addition, the necrotic cells are observed as red fluorescence.

**Figure 15:**
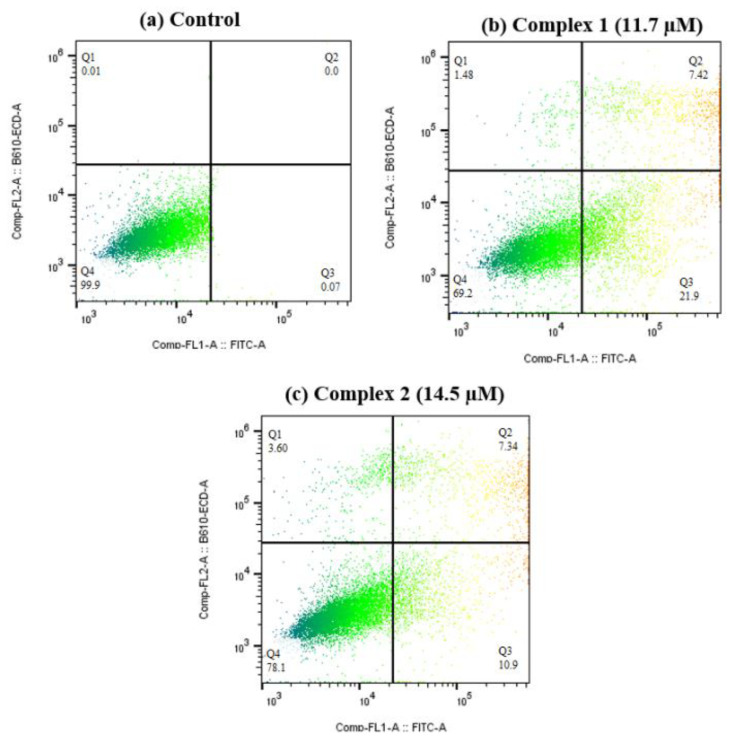
Flow cytometric analysis of control (**a**) live T24 cells; cells undergoing apoptosis: (**b**) cells treated with **1** and (**c**) cells treated with **2** at their respective IC_50_ concentrations. Quadrants: Q1, dead cells; Q2, late apoptotic cells; Q3, early apoptotic cells; Q4 live cells.

Quantitative assessment of early and late apoptosis in T24 cells induced by **1** and **2** at IC_50_ concentration was attained using flow cytometry coupled with fluorescein isothiocyanate (FITC) conjugated Annexin V assay. As show in Figure 15, **1** achieves an apoptotic rate of 29.32% (7.42% late and 21.9% early apoptosis) and **2** achieves an apoptotic rate of 18.24% (7.34% late and 10.9% early apoptosis), whereas the control displays negligible cell apoptosis. Overall, fluorescence imaging and flow cytometry analysis demonstrate that **1** and **2** effectively penetrate the membrane and promote T24 cell death through the apoptotic pathway, mainly inducing early apoptosis of T24 cells at IC_50_ concentration [39].

## 3. Discussion

Table 5 summarizes the trends of the four spectroscopic factors characterizing **1**–**7** and compares them with the trend of T24 IC_50_ values, wherein the trends for Log *p* and Ru–Cl bond length describe how easily a complex penetrates the cell membrane and becomes activated to interact with intracellular targets, respectively, and the binding affinity of the complex to DNA or protein is described by the binding constant. DFT calculations support trends in Ru–Cl bond length and Log *p*, and molecular docking studies support binding potencies to CTDNA and BSA. Although biophysicochemical studies of complex interaction with CTDNA or BSA reveal suitable properties of **1**–**7** as anticancer agents, the antiproliferation trend in T24 cells shows little correlation with the CTDNA or BSA binding potency trends. This means that at least all four factors synergistically affect the inhibitory activity on T24 cells. 

Anticancer activity depends on many factors, including Log *p*, Ru–Cl bond length, and DNA and protein binding abilities investigated in this study. These are commonly measured spectroscopic quantities, but the quantitative contribution of their respective antiproliferative effects remains an unexplored topic. It is possible that many factors are interdependent; however, we regard the simplest first-order approximation as the most straightforward and obvious approach, in the absence of established second-order interdependencies. A linear system of equations AX = b for regression is shown below:

$$\left[\begin{array}{cccc} 1 & 1 & 0 & 0\\ 0 & 0.60 & 0.48 & 1\\ 0.38 & 0.62 & 0.64 & 0.07\\ 0.58 & 0.17 & 0.75 & 0.10\\ 0.93 & 0.43 & 0.14 & 0.01\\ 0.12 & 0 & 0.93 & 0.02\\ 0.34 & 0.50 & 1 & 0.22 \end{array}\right]\left[\begin{array}{l} X_{LogP}\\ X_{Ru-Cl}\\ X_{bcDNA}\\ X_{bcBSA} \end{array}\right]=\left[\begin{array}{c} 1\\ 0.73\\ 0.37\\ 0.93\\ 0.14\\ 0\\ 0.43 \end{array}\right]$$

Entities in each row of A (7-by-4) are, in turn, the normalized Log *p*, Ru–Cl bond length, CTDNA and BSA binding constants for each complex. Entities in B (7-by-1) are the reciprocal of T24 IC_50_ values for each complex and normalized. The method of ordinary least squares is used to find an approximate solution X (4-by-1) to the overdetermined system of linear equations. The solutions are expressed as percentage of contributions, with Log *p*, Ru–Cl bond length, binding constants of CTDNA and BSA being 34.2%, 21.8%, 6.6%, and 37.3%, respectively. The solution suggests that Log *p* and binding to proteins are roughly equally important and collectively contribute 71.5%, while binding to nucleus DNA is the least important in the antiproliferative context of T24 cells. The solution also explains why **1** has the lowest binding affinity to both CTDNA and BSA, but is the most cytotoxic to T24 cells. Complex **1** most easily penetrates the cell membrane (highest Log *p*) and is most easily activated (longest Ru–Cl bond) to interact with intracellular targets with a total contribution of 56%, thereby promoting the best antiproliferation activity of **1** on T24 cells. Ru–Cl bond length may modulate the chloride-leaving ability for hydrolysis yielding the active Ru-OH_2_ species responsible for anticancer activity. Based on the first-order approximated statistical analysis of the experimental data, it was found that the contribution of Ru–Cl bond length to the anti-proliferation of T24 cells is 21.8%. It should also be noted that too rapid a rate of hydrolysis may not be beneficial as it may lead to adventitious side reactions.

## 4. Material and Methods

### 4.1. Material and Methods

Milli-Q Ultrapure water was used in all experiments. RuCl_3_.3H_2_O, 2,2′-bipyridine-4,4′-dicarboxlic acid (**L2**), CT-DNA, Bovine serum albumin (BSA), Ethidium bromide (EB) and Tris were purchased from Sigma Aldrich. All the other reagents and solvents (>96%) were received from various suppliers and used without further purification. The melting points were measured in open capillary tubes on a Lab India instrument. Electronic absorption spectra were recorded on Shimadzu UV-2550. Emission spectra were recorded on an Edinburgh FLS 1000 spectrofluorometer. FT-IR spectra of samples embedded in KBr pellets were obtained using a PerkinElmer spectrometer. NMR spectra were recorded in CDCl_3_/CD_3_OD/DMSO-d_6_ by using TMS as an internal standard on a Bruker 400 MHz spectrometer. MALDI-TOF spectra of complexes were recorded on a Bruker-Daltonics Omniflex spectrometer. EPR spectra were recorded on a Bruker EMX spectrometer equipped with a TE102 cavity. Further details on data fitting and analysis are provided in the supporting information (SI). Cell lines were obtained from Laboratory of Molecular Biology, Nanomedicine and Genomics, Faculty of Medicine, University of Atacama.

The shake-flask method was used to measure Log *p* of **1**–**7**. n-octanol and water containing 0.45% of sodium chloride were mutual saturated by stirring at 250 rpm for 24 h. The complex was dissolved in a mixture with equal volumes of pre-saturated n-octanol and water to a final concentration of 0.5 mM. The mixture was shaken at 250 rpm for 24 h at 37 °C and then centrifuged at 3000 rpm for 10 min. The complex concentration (C) in each phase was measured by UV-Vis spectroscopy and used to calculate Log *p*, according to the formula Log *p* = log (C_octanol_/C_water_).

All calculations were performed with the Gaussian 09 suite of programs [40] at the density functional theory (DFT) level of theory using the B3LYP, M06-L, and ωB97XD, functionals with mixed basis set. The Stuttgart–Dresden relativistic effective core potential and its associated basis sets (SDD) [41] was used for constructing the molecular orbitals of Ru and the 6-31+G(d,p) for all other atoms. The universal SMD implicit solvation model [42] was used to describe the n-octanol and water solvent effects. During the total-energy minimization, all atoms were allowed to relax without constraints. Frequency calculations were performed for the optimized minimum-energy structures to verify that they correspond to equilibrium stationary minima and to obtain corrections to the free energies. The corrected Gibbs energies obtained in water ($G_{wat}^{0}$) and in dry or wet n-octanol ($G_{oct}^{0}$) at 298.15 K were used to calculate Log *p* according to:

(1) $$Log\;P=-\frac{∆G_{trans}^{0}}{RTln10}=-\frac{G_{oct}^{0}-G_{wat}^{0}}{RTln10}$$

where $∆G_{trans}^{0}$ is the Gibbs energy of transfer between the two solvents, R is the molar gas constant, and T is the temperature (298.15 K). Additionally, Log *p* is calculated by the methodology developed by Molinspiration as a sum of fragment-based contributions and correction factors, and trained on more than 12,000 molecular structures [25].

GaussView 5.0 was used to plot molecular properties [43]. GaussSum 3.0 was used to plot atom projected density of states and crystal orbital overlap populations [21].

The docking simulations were conducted using AutoDock version 4.2.6.1 and AutoDock Tools version 1.5.6 obtained from the Scripps Research Institute (La Jolla, CA, USA) [44]. The bond lengths and angles of **1**–**7** were taken from the structures optimized in gas phase at the B3LYP level using the Gaussian 09 suite of programs. All possible torsions were set free to perform flexible ligand docking. The size of the search space was large enough to accommodate the site of a fully extended Ru(II)-complex and its interacting residues. One hundred structures were generated using the Lamarckian genetic algorithm. The number of energy evaluations was set to a maximum of 25,000,000 runs. The lowest binding energy mode of the most populated conformationally similar cluster is considered as the best docking mode.

### 4.2. Synthesis & Characterization of Ligands (L1–L4)

#### 4.2.1. 2,2′-Bipyridine-4,4′-di-tert-butyl Ligand (L1)

The ligand was synthesized as described previously [45]. A round-bottom flask was charged with sodium amide (4.4 g, 0.338 mmol) and 50 mL 4-tert-butylpyridine under argon. The temperature was raised to 135–140 °C and maintained for 6 h, when a metallic purple color was observed. The boiling point of 4-tert-butylpyridine is too high for the reflux reaction to be practical. Consequently, the reaction mixture was cooled to around 40 °C to afford a dark green colored solution. After this observation, the reaction mixture was extracted with 100 mL of xylene, washed with 20 mL of water to remove the unreacted, excess sodium amide. The organic layers were collected and dried over Na_2_SO_4_. Finally, the mixture was distilled under reduced pressure at 60 °C to remove the solvent (xylene) and unreacted 4-tert-butylpyridine. The obtained yellow crude product was recrystallized using hot acetone. The title compound was crystallized as white needles (3.7 g, 13.9 mmol). L1 white solid. Yield: 72%. M.p.: 160 °C. UV-Vis (CH_3_OH): λmax, nm (ε, dm^3^ mol^−1^ cm^−1^) 240 (73,950), 298 (79,670). ^1^H NMR (400 MHz, CDCl_3_): δ, ppm 8.59 (d, J = 5.2 Hz, 2H), 8.40 (d, J = 1.5 Hz, 2H), 7.30 (dd, J = 5.2, 1.9 Hz, 2H), 1.38 (s, 18H).

#### 4.2.2. 2,2′-Bipyridine-4,4′-Dicarboxylic Acid Ligand (L2)

L2 white solid. M.p.: 316 °C. UV-Vis (DMSO): λ_max_, nm (ε, dm^3^ mol^−1^ cm^−1^) 251 (27,890), 298 (79,150). ^1^H NMR (400 MHz, DMSO-d_6_): δ, ppm 8.95 (d, J = 4.8 Hz, 2H), 8.88 (s, 1H), 7.95 (d, J = 4.3 Hz, 2H).

#### 4.2.3. Dimethyl or Diethyl 2,2′-bipyridine-4,4′-Dicarboxylate Ligand (L3) or (L4)

4,4′-dicarboxy-2,2′-bipyridine was dissolved in absolute methanol or ethanol under nitrogen atmosphere in a 100 mL round-bottom flask. The mixture was stirred at 0 °C for 20 min, while concentrated H_2_SO_4_ was added. During this addition, a pale pink color was observed. The reaction temperature was then increased to reflux and continued for several hours. Then, the mixture was poured into ice water (300 mL) to obtain a white precipitate of the L3 or L4 compound. The precipitate was filtered, washed with water, and dried under vacuum. The pure crystal of the compound was obtained from methanol and acetonitrile solvent mixture in 1:1 ratio. **L3** white solid Yield: 96%. UV-Vis (CH_3_OH): λ_max_, nm (ε, dm^3^ mol^−1^ cm^−1^) 240 (51,690), 292 (56,290)), ^1^H NMR (400 MHz, CDCl_3_): δ, ppm 8.96 (s(broad), 2H), 8.87 (d, *J* = 4.9 Hz, 2H), 7.90 (d, *J* = 4.9, 1.5 Hz, 2H), 4.00 (s,6H). **L4** white solid Yield: 95 %. UV-Vis (CH_3_OH): λ_max_, nm (ε, dm^3^ mol^−1^ cm^−1^) 240 (67,720), 291 (73,950), ^1^H NMR (400 MHz, CDCl_3_): δ, ppm 8.94 (broad, 2H), 8.87 (d, *J* = 5.0 Hz, 2H), 7.91 (dd, *J* = 4.9, 1.5 Hz, 2H), 4.46 (q, *J* = 7.1 Hz, 4H), 1.44 (t, *J* = 7.1 Hz, 6H).

### 4.3. Procedure for the Preparation of Complexes ***1***–***7***

[(η^6^-*p*-cymene)-Ru^II^Cl(µ-Cl)]_2_ was synthesized per the reported method [46]. [(η^6^-*p*-cymene)-Ru^II^Cl(µ-Cl)]_2_ dimer 0.1224 g (0.2 mmol) and ligands **L1**, **L3,** or **L4** were dissolved in 25 mL of CH_2_Cl_2_ and stirred for 6−8 h at room temperature. The dark red color solution was concentrated to ∼5 mL under reduced pressure, and addition of excess hexane gave a clear orange solid. The product was collected by filtration, washed with petroleum ether, and dried in vacuo to obtain products **1**, **3,** and **4**. Complex **2** was prepared by modification of a literature procedure [47]. A 0.2 mmol (0.1224 g)[RuCl(µ-Cl)(η^6^-p-cymene)]_2_ and 2,2′-bipyridine-4,4′-dicarboxylic was added in 10 mL of anhydrous DMF. The reaction mixture was purged with argon for 5 min and then stirred to reflux conditions at 80 °C for 4 h under argon. During the reaction course, a dark red color was observed. After cooling to RT, it was poured into ice water 200 (mL) and allowed for several hours to settle down precipitate at bottom of the solution. Then, the solvent layer was gently decanted, and the obtained product was washed with water and dried under vacuum. The obtained yellow or orange product was used without further purification. The observed ^1^H NMR characterization data are in good agreement with literature values [48].

[(η^6^-Benzene)-Ru^II^Cl(μ-Cl)]_2_ was synthesized per reported procedures [49]. The prepared Ru(II)-benzene dimer (0.100 g, 0.2 mmol) was added in 20 mL of CH_2_Cl_2_ and sonicated for 15 minutes in a round-bottom flask. The dissolved appropriate ligands 20 mL (**L3** or **L4**) were added to the reaction mixture, stirred to obtain a clear solution, and sequentially refluxed for 8–10 h. The obtained clear red solution was concentrated to ∼2 mL under reduced pressure, and by addition of excess of hexane an orange colored solid compound was obtained. The solid was collected by filtration, washed with hexane several times and air dried to obtain products **6** and **7**. Complex **5** was synthesized using the reported method [50].

### 4.4. Characterization of Complexes ***1***–***7***

2.4.1 [Ru-η^6^-p-cymene-(4,4′-(C_4_H_9_)_2_bipy)Cl]Cl (1). L1 (0.1072 g, 0.4 mmol) was used. Orange solid. Yield: 91%. M.p.: 210 °C. Anal. Calc. for C_28_H_38_Cl_2_N_2_Ru: C, 58.53; H, 6.67; N, 4.88. Found: C, 59.08; H,6.65; N, 4.76. MALDI-MS (m/z): [M−Cl]^+^ = 539.1823. UV-Vis (water): λ_max_, nm (ε, dm^3^ mol^−1^ cm^−1^) 263 (54766), 303 (50720), 406 (4226). FT–IR (KBr, cm^−1^): ʋ(C=N); 1611 (s), ʋ(Ru–Cl) 740 (w). ^1^H NMR (400 MHz, CDCl_3_): δ, ppm 9.71 (d, J = 6.0 Hz, 2H), 7.96 (broad s, 2H), 7.73 (d, J = 5.9 Hz, 2H), 6.26 (d, J = 6.0 Hz, 2H), 6.14 (d, J = 6.0 Hz, 2H), 2.76–2.69 (m, 1H), 2.26 (s, 3H), 1.41 (sharp s, 18H), 1.06 (d, J = 6.9 Hz, 6H). 

2.4.2 [Ru-η^6^-p-cymene-(4,4′-(COOH)_2_bipy)Cl]Cl (2). **L2** (0.0976 g, 0.4 mmol) was used. Orange solid. Yield: 75%. M.p.: 198 °C. Anal. Calc. for C_22_H_22_Cl_2_N_2_O_4_Ru: C, 48.01; H, 4.03; N, 5.09. Found: C, 50.08; H, 4.02; N, 5.11. MALDI-MS (m/z) [M−Cl]^+^ = 515.045. UV-Vis (CH_3_OH): λ_max_, nm (ε, dm^3^ mol^−1^ cm^−1^) 263 (51,993), 288 (53,720), 403 (5153). FT–IR (KBr, cm^−1^): ʋ(C=O); 1732 (s), ʋ(C=N); 1626 (s), ʋ(Ru–Cl) 742 (w). ^1^H NMR (400 MHz, CD_3_OD) δ 9.61 (d, *J* = 6.4 Hz, 2H), 8.98 (s, 2H), 8.21 (dd, *J* = 5.2, 2.9 Hz, 2H), 6.18 (d, *J* = 6.2 Hz, 2H), 5.94 (d, *J* = 5.2 Hz, 2H), 2.68 (m, 1H), 2.29 (sharp s, 3H), 1.08 (d, *J* = 6.9 Hz, 6H).

2.4.3 [Ru-η^6^-p-cymene-(4,4′-(COOCH_3_)_2_bipy)Cl]Cl (3). **L3** (0.1088 g, 0.4 mmol) was used. Orange solid. Yield: 89%. M.p.: 164 °C. Anal. Calc. for C_24_H_26_Cl_2_N_2_O_4_Ru: C, 49.83; H, 4.53; N, 4.84. Found: C, 50.38; H, 4.51; N, 4.82. MALDI-MS (m/z) [M−Cl]^+^ = 543.0739. UV–Vis (water): λ_max_, nm (ε, dm^3^ mol^−1^ cm^−1^) 247 (50,720), 294 (46,820), 404 (8486). FT–IR (KBr, cm^−1^): ʋ(C=O); 1711 (s), ʋ(C=N); 1624 (s), ʋ(Ru–Cl) 766 (w). ^1^H NMR (400 MHz, CDCl_3_): δ, ppm 9.67 (d, *J* = 5.9 Hz, 2H), 8.64 (s, 2H), 8.31 (d, *J* = 5.7 Hz, 2H), 6.50 (d, *J* = 6.3 Hz, 2H), 6.32 (d, *J* = 6.3 Hz, 2H), 4.01 (q, *J* = 7.1 Hz, 6H), 2.77–2.68 (m, 1H), 2.28 (s, 3H), 1.02 (d, *J* = 6.9 Hz, 6H).

2.4.4 [Ru-η^6^-p-cymene-(4,4′-(COOC_2_H_5_)_2_bipy)Cl]Cl (4). **L4** (0.120 g, 0.4 mmol) was used. Orange solid. Yield: 93%. M.p.: 181 °C. Anal. Calc. for C_26_H_30_Cl_2_N_2_O_4_Ru: C, 51.49; H, 4.99; N, 4.62. Found: C, 52.08; H, 4.97; N, 4.59. MALDI-MS (m/z): [M−Cl]^+^ = 571.1001. UV-Vis (water): λ_max_, nm (ε, dm^3^ mol^−1^ cm^−1^) 248 (50,493), 303, (53,146), 406 (9986). FT–IR (KBr, cm^−1^): ʋ(C=O); 1717 (s), ʋ(C=N); 1625 (s), ʋ(Ru–Cl) 765 (w). ^1^H NMR (400 MHz, CDCl_3_): δ, ppm 10.14 (d, *J* = 5.9 Hz, 2H), 8.67 (s, 2H), 8.31 (d, *J* = 5.7 Hz, 2H), 6.47 (d, *J* = 6.3 Hz, 2H), 6.29 (d, *J* = 6.3 Hz, 2H), 4.51 (q, *J* = 7.1 Hz, 4H), 2.77–2.68 (m, 1H), 2.32 (s, 3H), 1.46 (t, *J* = 7.1 Hz, 6H), 1.05 (d, *J* = 6.9 Hz, 6H).

2.4.5 [Ru-η^6^-benzene-(4,4′-(C_4_H_9_)_2_bipy)Cl]Cl (5). **L1** (0.1072 g, 0.4 mmol) was used. Orange solid. Yield: 91%. M.p.: 193 °C. Anal. Calc. for C_24_H_30_Cl_2_N_2_Ru: C, 55.60; H, 5.83; N, 5.40. Found: C, 56.08; H, 5.84; N, 5.35. MALDI-MS (m/z): [M−Cl]^+^ = 482.7944. UV-Vis (water): λ_max_, nm (ε, dm^3^ mol^−1^ cm^−1^) 263 (55100), 304 (49813), 402 (3313). FT–IR (KBr, cm^−1^): ʋ(C=N); 1614 (s), ʋ(Ru–Cl) 742 (w). ^1^H NMR (400 MHz, CDCl_3_): δ, ppm 9.77 (d, *J* = 5.7 Hz, 2H), 7.97 (broad s, 2H), 7.70 (d, *J* = 5.8 Hz, 2H), 6.33 (sharp s, 6H), 1.42 (s, 18H).

2.4.6 [Ru-η^6^-benzene-(4,4′-(COOCH_3_)_2_bipy)Cl]Cl (6). **L3** (0.1088 g, 0.4 mmol) was used. Orange solid. Yield: 90%. M.p.: 206 °C. Anal. Calc. for C_20_H_18_Cl_2_N_2_O_4_Ru: C, 45.99; H, 3.47; N, 5.36. Found: C, 46.37; H, 3.45; N, 5.34. MALDI-MS (*m*/*z*): [M−Cl]^+^ = 487.0051. UV-Vis (water): λ_max_, nm (ε, dm^3^ mol^−1^ cm^−1^) 248 (49700), 288 (47274), 403 (6533). FT–IR (KBr, cm^−1^): ʋ(C=O); 1731 (s), ʋ(C=N); 1623 (s), 758 (w). ^1^H NMR (400 MHz, CD_3_OD): δ, ppm 9.78 (d, 2H), 9.06 (sharp s, 2H), 8.24 (d, 2H), 6.24 (sharp s, 6H), 4.10 (s, 6H).

2.4.7 [Ru-η^6^-benzene-(4,4′-(COOC_2_H_5_)_2_bipy)Cl]Cl (7). **L4** (0.120 g, 0.4 mmol) was used. Orange solid. Yield: 92%. M.p.: 210 °C. Anal. Calc. for C_22_H_22_Cl_2_N_2_O_4_Ru: C, 48.01; H, 4.03; N, 5.09. Found: C, 50.01; H, 3.99; N, 5.02. MALDI-MS (*m*/*z*): [M−Cl]^+^ = 514.7866. UV-Vis (water): λ_max_, nm (ε, dm^3^ mol^−1^ cm^−1^) 247 (47730), 285 (42326), 403 (5720). FT–IR (KBr, cm^−1^): ʋ(C=O); 1724 (s), ʋ(C=N); 1620 (s), 764 (w). ^1^H NMR (400 MHz, CDCl_3_): δ, ppm 10.36 (broad s, 2H), 8.62 (sharp s, 2H), 8.27 (broad s, 2H), 6.56 (sharp s, 6H), 4.48 (q, *J* = 7.0 Hz, 4H), 1.44 (t, *J* = 7.1 Hz, 6H).

## 5. Conclusions

The main value of this work lies in the synthesis of a series of novel Ru(II)-ɳ^6^-arene complexes with 4,4′-para-substituted 2,2′-bipyridine ligands that exhibit in vitro antiproliferative activity against human bladder cancer T24 cells more than three times higher than cisplatin. Of particular interest is that the characteristics of **1** and **2** as anticancer candidates for T24 have excellent practical values in both cytotoxicity and specific selectivity, which is significant as the common limitation of most metal complexes as anticancer drugs in clinical use is their general toxicity. DFT computations, PDOS and COOP analyses reveal structural (Ru–Cl bond length) and thermodynamic (Log *p*) properties of **1**–**7** that are fully consistent with the experimental observations. Spectroscopic and molecular docking results are consistent with each other and collectively show that subtle structural changes of the 2,2′-bipyridine ligand alter their binding poses and affinities to CTDNA or BSA. Fluorescence imaging and flow cytometry analysis demonstrate that **1** and **2** effectively penetrate the membrane and promote T24 cell death through the apoptotic pathway, mainly inducing early apoptosis of T24 cells at IC_50_ concentration. Statistical analysis suggests that Log *p* and binding to proteins are roughly equally important and collectively contribute more than 70%, while binding to nucleus DNA is the least important in the antiproliferative context of T24 cells.

Although we have shown that the ability (Log *p*) of these complexes to penetrate the lipid bilayer of the cell membrane contributes most significantly (34.2%) to the T24 anticancer activity, the specific mechanism by which these complexes operate at the cellular level to induce early apoptosis in T24 cells has not been resolved yet; however, this study initiated and explored the in vitro anticancer activity of Ru-arene complexes against human bladder cancer cells for the first time, and achieved promising results. Future pharmacodynamical studies may shed light on the specific intracellular mechanism and lead to the rational design of novel Ru-arene complexes with in vivo selective cytotoxicity for practical clinical evaluation.

## Figures and Tables

**Table 1:** Calculated gas phase Ru–Cl bond length and water-octanol Log *p* values using different DFT functionals, and comparison with experimental values.

	Ru–Cl Bond Length (Å)				Log *p*					
	Exp.	DFT Theory (Gas Phase)			Exp.	Fragment-Based	DFT Theory (SMD Implicit Solvation Model)			
						Calculator	n-Octanol			Water-Saturated n-Octanol
Complex	XRD	B3LYP	M06-L	ωB97XD	Shake Flask	miLogP	B3LYP	M06-L	ωB97XD	ωB97XD
**1**	2.423	2.403	2.411	2.389	2.75	6.68	6.060	5.868	5.698	4.506
**2**	2.406	2.400	2.404	2.385	−1.65	3.09	−0.872	−0.207	−0.849	−2.677
**3**	2.407	2.401	2.411	2.388	0	3.61	2.775	1.662	1.671	−0.248
**4**	2.388	2.402	2.412	2.388	0.88	4.36	3.492	3.528	3.441	1.956
**5**	2.399	2.395	2.401	2.380	2.45	5.04	4.073	5.137	4.671	3.385
**6**	2.381	2.392	2.396	2.377	−1.12	1.97	0.083	0.575	0.336	−1.137
**7**	2.402	2.391	2.392	2.376	−0.14	2.73	1.642	2.027	1.972	0.582
	MAD ^a^	0.010	0.010	0.018	MSD ^b^	3.47	2.01	2.20	1.96	0.46

^a^ MAD: mean absolute deviation; ^b^ MSD: mean signed deviation.

**Table 2:** Experimental CTDNA binding (K_b_), Stern–Volmer (K_q_), apparent binding (K_app_) constants and calculated free energy of binding (ΔG) through molecular docking analysis.

	Experiments			Docking
Comp.	*K*_b_ (10^4^ M^−1^)	*K*_q_ (10^4^ M^−1^)	*K*_app_ (10^6^ M^−1^)	ΔG (kcal/mol)
**1**	1.12	3.65	1.83	−6.10
**2**	1.40	4.89	3.00	−6.46
**3**	1.50	6.05	3.02	−6.94
**4**	1.56	6.36	3.18	−7.23
**5**	1.20	3.84	1.92	−6.19
**6**	1.67	6.69	3.34	−7.87
**7**	1.71	7.21	3.61	−8.36

**Table 3:** Experimental BSA binding (Kb), quenching (Kq) constants, number of binding sites (n), and calculated free energy of binding (ΔG) for **1**–**7**.

	Experiments			Docking
Complex	*K*_b_ (10^4^ M^−1^)	*K*_q_ (10^4^ M^−1^)	*n*	ΔG (kcal/mol)
**1**	9.335 × 10^3^	2.145 × 10^4^	0.9	−6.91
**2**	5.757 × 10^5^	6.446 × 10^4^	1.2	−8.12
**3**	5.071 × 10^4^	3.622 × 10^4^	1.0	−7.36
**4**	6.354 × 10^4^	3.777 × 10^4^	1.0	−7.39
**5**	1.132 × 10^4^	2.587 × 10^4^	0.9	−7.41
**6**	2.204 × 10^4^	3.103 × 10^4^	1.0	−7.11
**7**	1.341 × 10^5^	4.779 × 10^4^	1.1	−7.49

**Table 4:** IC_50_ values of **1**–**7** and cisplatin against MCF-10a, Vero, EA.hy926, HepG-2, and T24 cells.

Comp.	MCF-10a	Vero	EA.hy926	HepG-2	T24
**1**	48.5	>50	>50	30.1	11.7
**2**	>50	>50	>50	>50	14.5
**3**	>50	45.6	30.8	>50	21.2
**4**	40.1	31.2	32.3	21.1	12.3
**5**	>50	>50	>50	>50	30.5
**6**	>50	49.2	49.3	14.5	41.2
**7**	>50	33.9	23.8	41.6	19.8
Cisplatin	29.1	33.7	22.9	49.1	>50

**Table 5:** Comparison of spectroscopic trends with the T24 antiproliferation trend.

Observations	Order	Origin
IC50 values against T24 cells	**1** < **4** < **2** < **7** < **3** < **5** < **6**	Table 4
Log *p*	**1** > **5** > **4** > **3** > **7** > **6** > **2**	Table 1
Ru–Cl bond length	**1** > **3** ≥ **2** > **7** > **5** > **4** > **6**	Table 1
CT DNA binding constant	**7** > **6** > **4** > **3** > **2** > **5** > **1**	Table 2
BSA binding constant	**2** >> **7** > **4** > **3** > **6** > **5** > **1**	Table 3

## Data Availability

The data presented in this study are available on request from the corresponding author.

## Supplementary Materials

The supporting information can be downloaded at: https://www.mdpi.com/article/10.3390/ijms241511896/s1, Experimental details; data fitting and analysis; NMR spectra, crystal data and refinements; MTT assay; CTDNA and BSA binding and docking data; DNA fragmentation; literature data.
