# Supplementary material for: Effective and Selective Ru(II)-Arene Complexes Containing 4,4′-Substituted 2,2′ Bipyridine Ligands Targeting Human Urinary Bladder Cancer Cells
Source: Int J Mol Sci. 2023 Jul 25;24(15):11896. doi: 10.3390/ijms241511896 (PMC10418970; doi:10.3390/ijms241511896)
Supplement: Supplementary file 1 [file ijms-24-11896-s001.zip › ijms-2505261-supplementary.pdf]

Effective and Selective Ru(II)-Arene Complexes Containing 4,4′-Substituted 2,2′ Bipyridine Ligands Targeting Human Urinary Bladder Cancer Cells

Mathiyan Muralisankar 1, Jun-Ru Chen 1, Jebiti Haribabu 2 and Shyue-Chu Ke 1,*

1 Physics Department, National Dong Hwa University, Hualien 97401, Taiwan; murali6289@gmail.com (M.M.); 410114237@gms.ndhu.edu.tw (J.-R.C.)

2 Facultad de Medicina, Universidad de Atacama, Copayapu 485, Copiapo 1531772, Chile; haribabusiri970@gmail.com

* Correspondence: ke@gms.ndhu.edu.tw

1. Single crystal X-ray diffraction studies

A Leica MZ 75 microscope was used to identify faces with dimensions of representative sample crystal. The crystal was mounted on a tip of glass fiber coated in paratone oil and then placed in a cold nitrogen stream (Oxford) maintained at 100 K. Bruker APEX II CCD diffractometer was employed for crystal screening, unit cell determination, and data collection. The X-ray radiation employed was generated from a Mo sealed X-ray tube (Kα = 0.70173Å with a potential of 40 kV and a current of 40 mA). Integrated intensity information for each reflection was obtained by reduction of the data frames with the program APEX2. The integration method employed a three-dimensional profiling algorithm and all data were corrected for Lorentz and polarization factors, as well as for crystal decay effects. Finally, the data were merged and scaled to produce a suitable data set. The absorption correction program SADABS was employed to correct the data for absorption effects. The structure was solved by direct methods and refined by least-squares against F^2 using the SHELXL-2018/3 package, incorporated in SHELXTL/PC V6.14. The H atoms were geometrically fixed and set riding on the corresponding parent atoms. The final data representation and structure plots were acquired by Olex.

2. Solution behaviour of the complexes (1-7) in PH 7.2 Tris-buffered solution at NaCl concentration of 4 mM and 110 mM

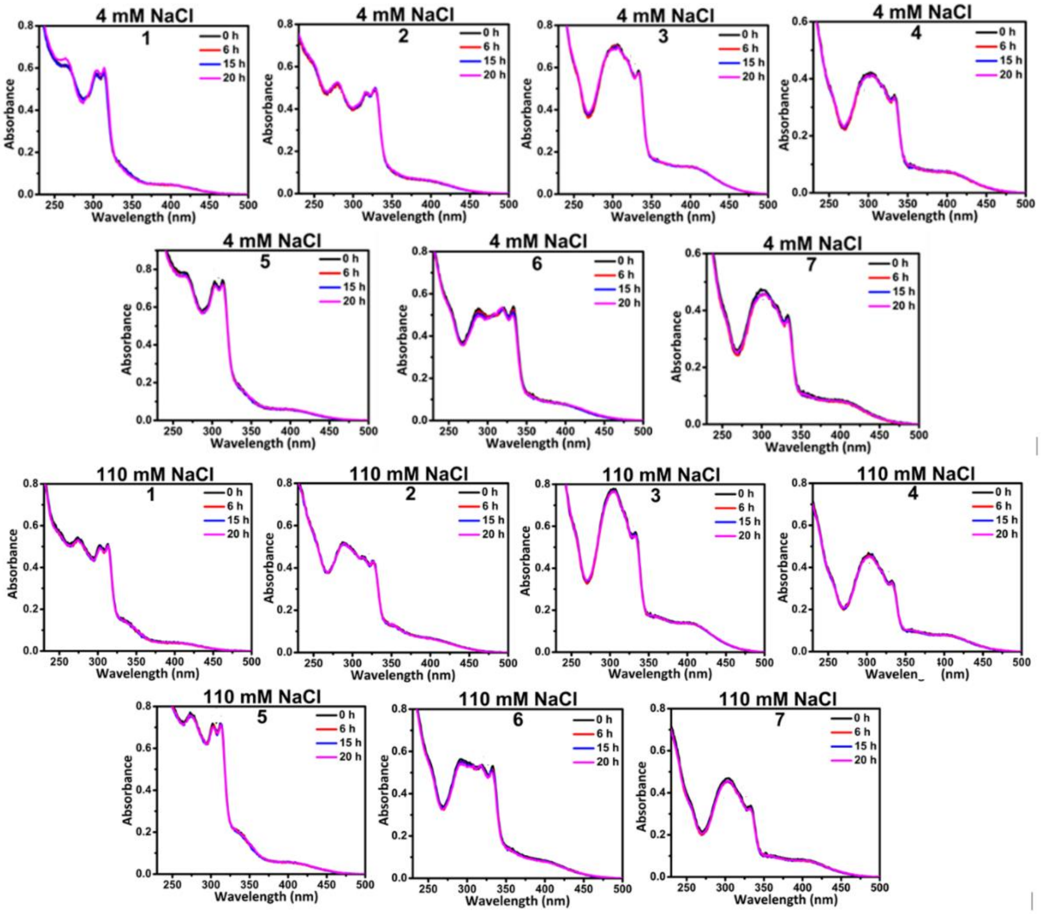

Figure S1. Time-dependent UV-Vis spectra measuring the stability of the complexes (**1-7**) in PH 7.2 Tris-buffered solution at NaCl concentration of 4 mM and 110 mM.

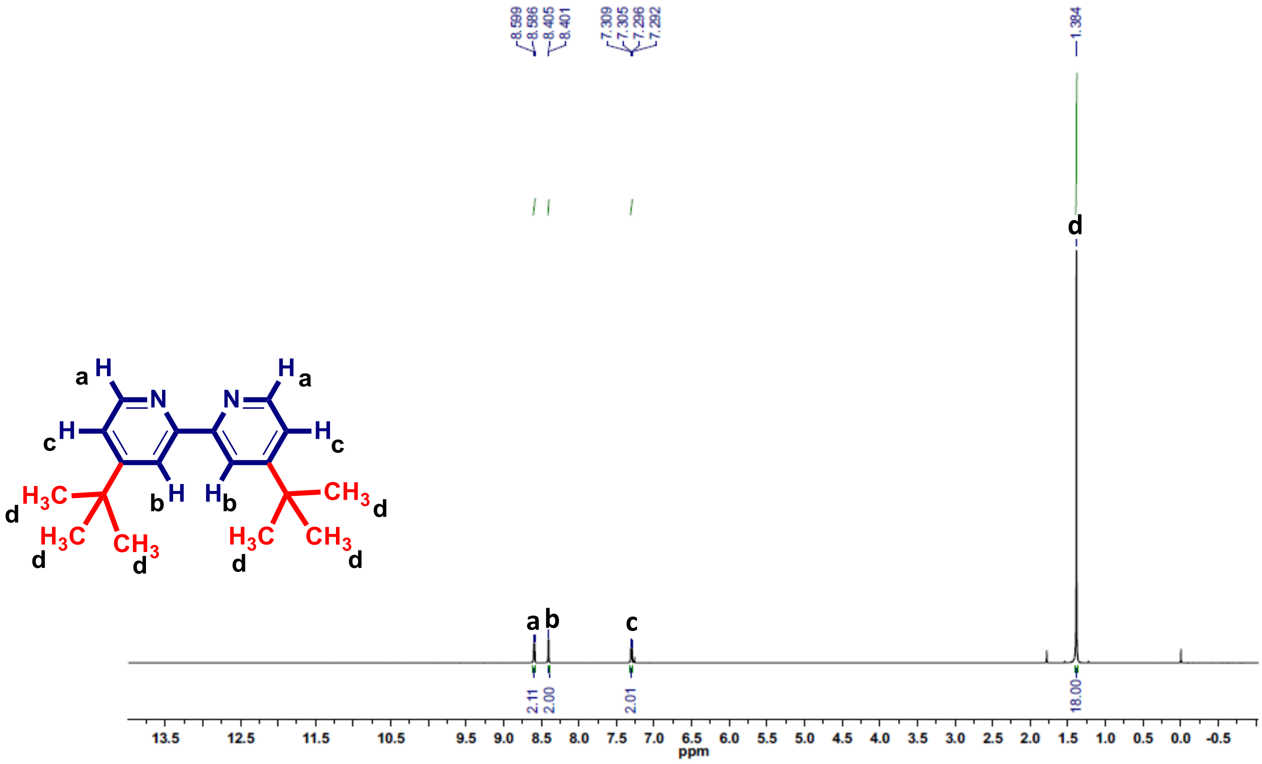
8.599
8.596
8.405
8.401
7.309
7.305
7.296
7.292
1.384
d
a H
N
N
H a
c H
H c
H₃C
b H
H b
CH₃ d
d
H₃C
CH₃
H₃C
CH₃
d
d
d
d
a b
c
2.11
2.00
2.01
18.00
13.5 12.5 11.5 10.5 9.5 9.0 8.5 8.0 7.5 7.0 6.5 6.0 5.5 5.0 4.5 4.0 3.5 3.0 2.5 2.0 1.5 1.0 0.5 0.0 -0.5
ppm

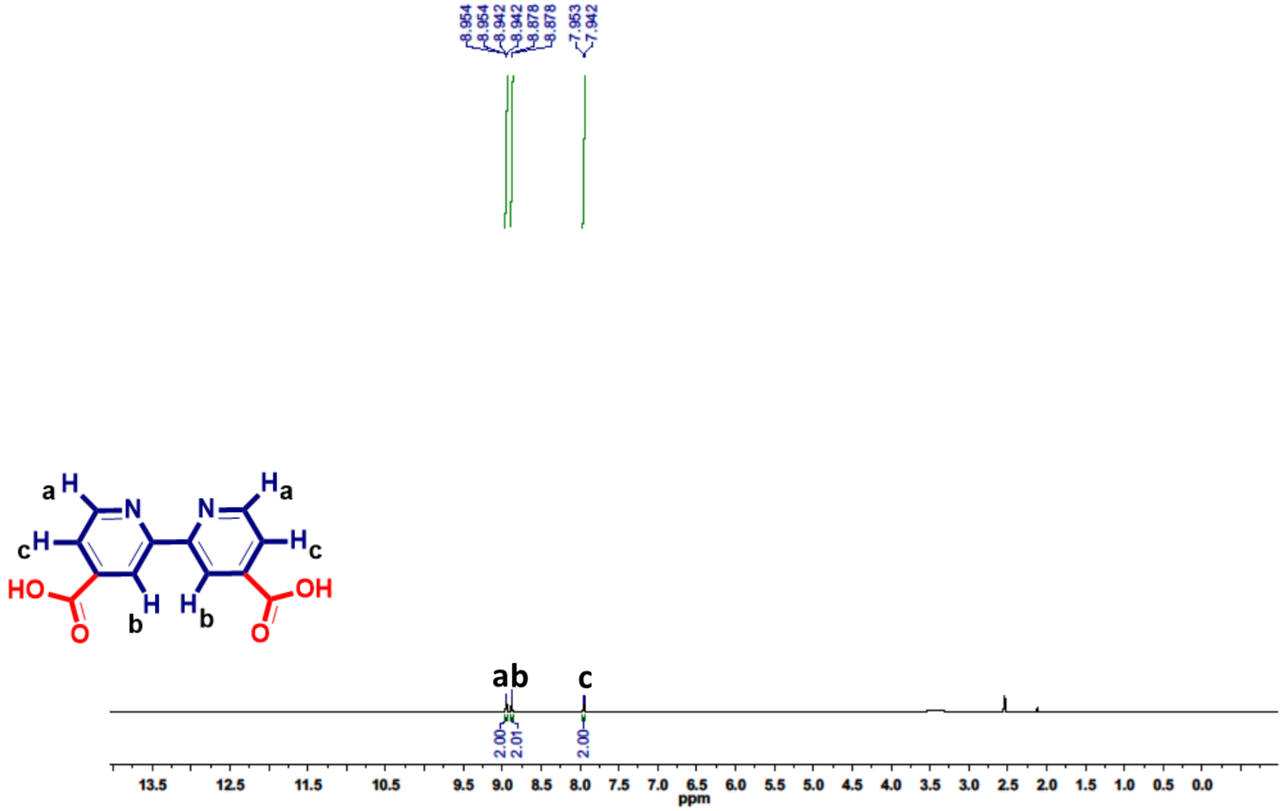
8.954
8.954
8.942
8.942
8.878
8.878
7.953
7.942
a H
N
N
H a
c H
H c
HO
O
H
b
H b
OH
O
ab
c
2.00
2.01
2.00
13.5 12.5 11.5 10.5 9.5 9.0 8.5 8.0 7.5 7.0 6.5 6.0 5.5 5.0 4.5 4.0 3.5 3.0 2.5 2.0 1.5 1.0 0.5 0.0
ppm

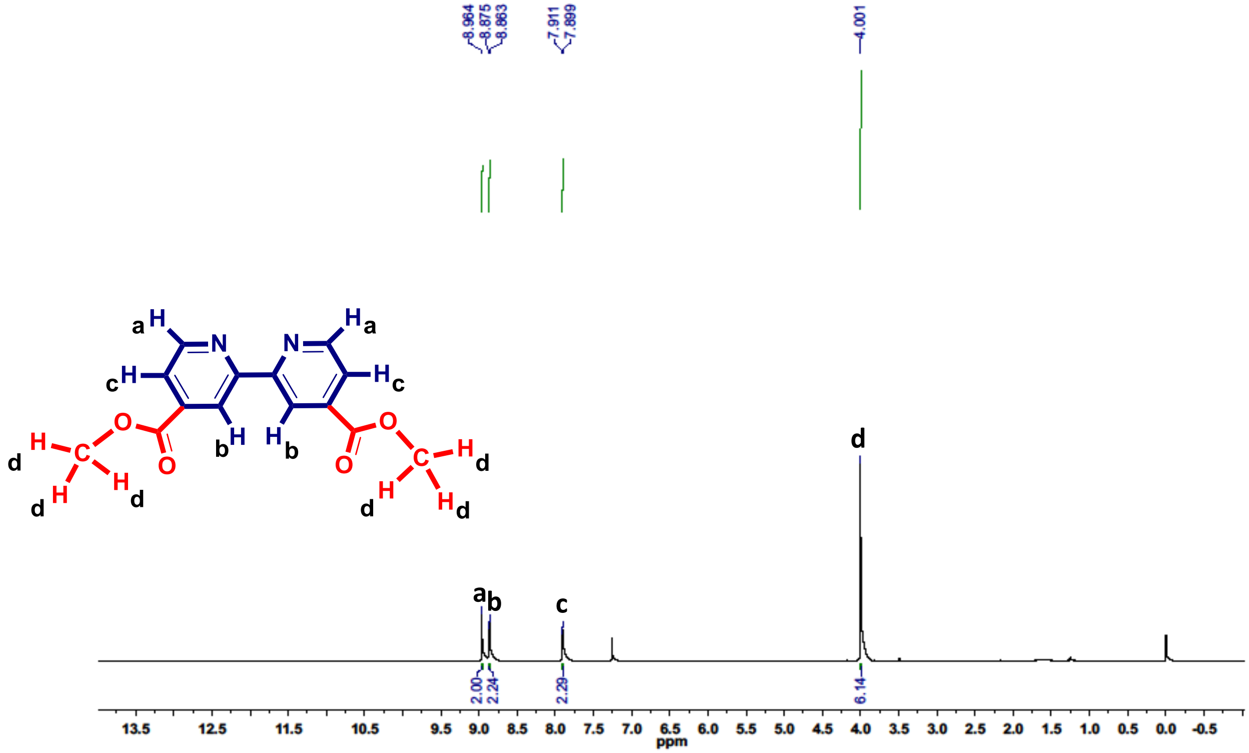

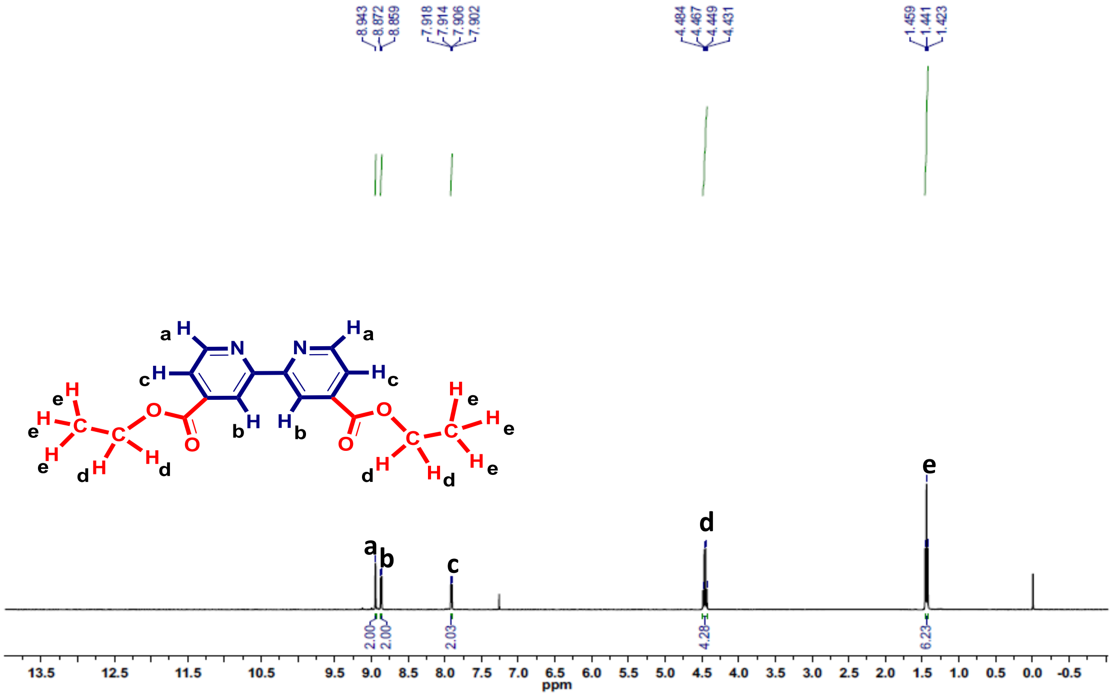

Figure S2. ^{1}H NMR spectra of ligands (L1-L4)

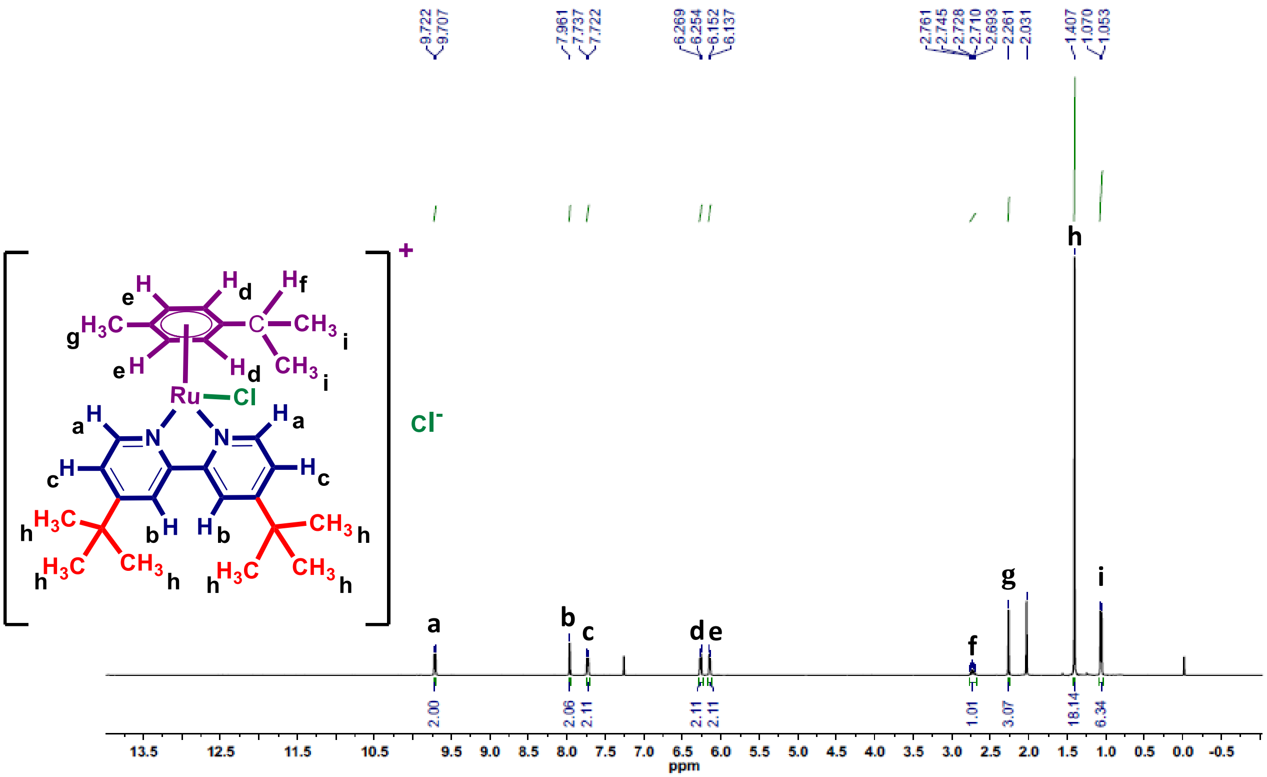
9.722
9.707
7.961
7.737
7.722
6.269
6.254
6.152
6.137
2.761
2.745
2.728
2.710
2.693
2.261
2.031
1.407
1.070
1.053
Ru
Cl
N
Cl-
a
b
c
d e
f
g
h
i
2.00
2.06
2.11
2.11
2.11
1.01
3.07
18.14
6.34
13.5
12.5
11.5
10.5
9.5
9.0
8.5
8.0
7.5
7.0
6.5
6.0
5.5
5.0
4.5
4.0
3.5
3.0
2.5
2.0
1.5
1.0
0.5
0.0
-0.5
ppm

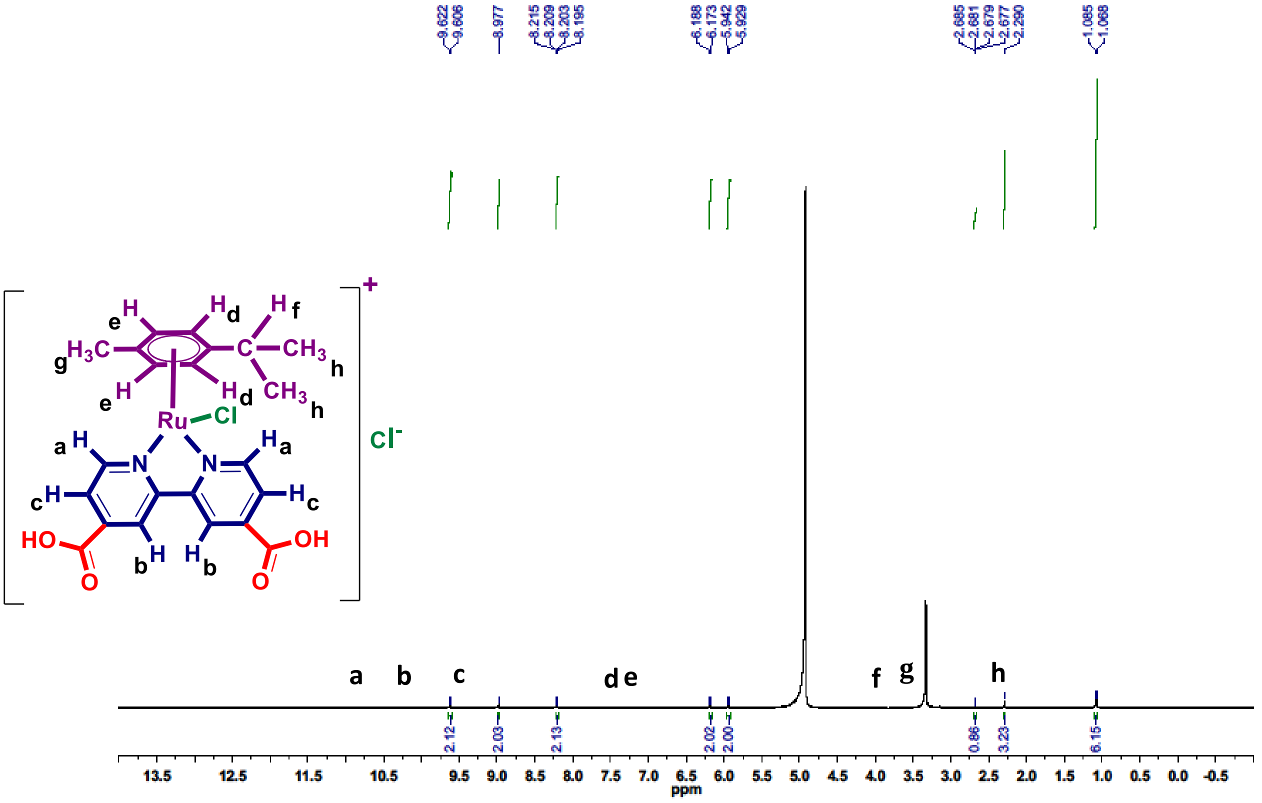
9.622
9.606
8.977
8.215
8.209
8.203
8.195
6.188
6.173
5.942
5.929
2.685
2.681
2.679
2.677
2.290
1.085
1.068
Ru
Cl
N
HO
OH
O
Cl-
a
b
c
d e
f
g
h
2.12
2.03
2.13
2.02
2.00
0.86
3.23
6.15
13.5
12.5
11.5
10.5
9.5
9.0
8.5
8.0
7.5
7.0
6.5
6.0
5.5
5.0
4.5
4.0
3.5
3.0
2.5
2.0
1.5
1.0
0.5
0.0
-0.5
ppm

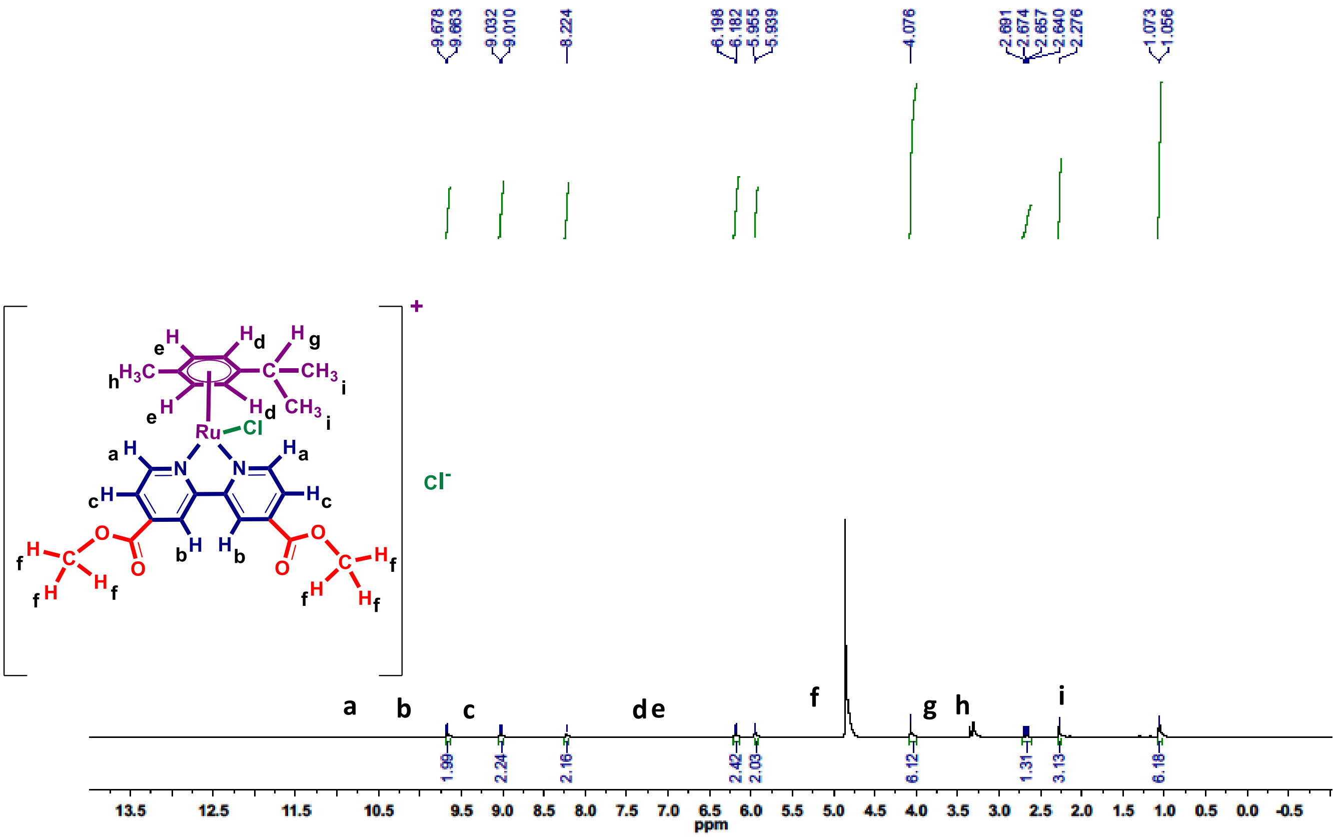

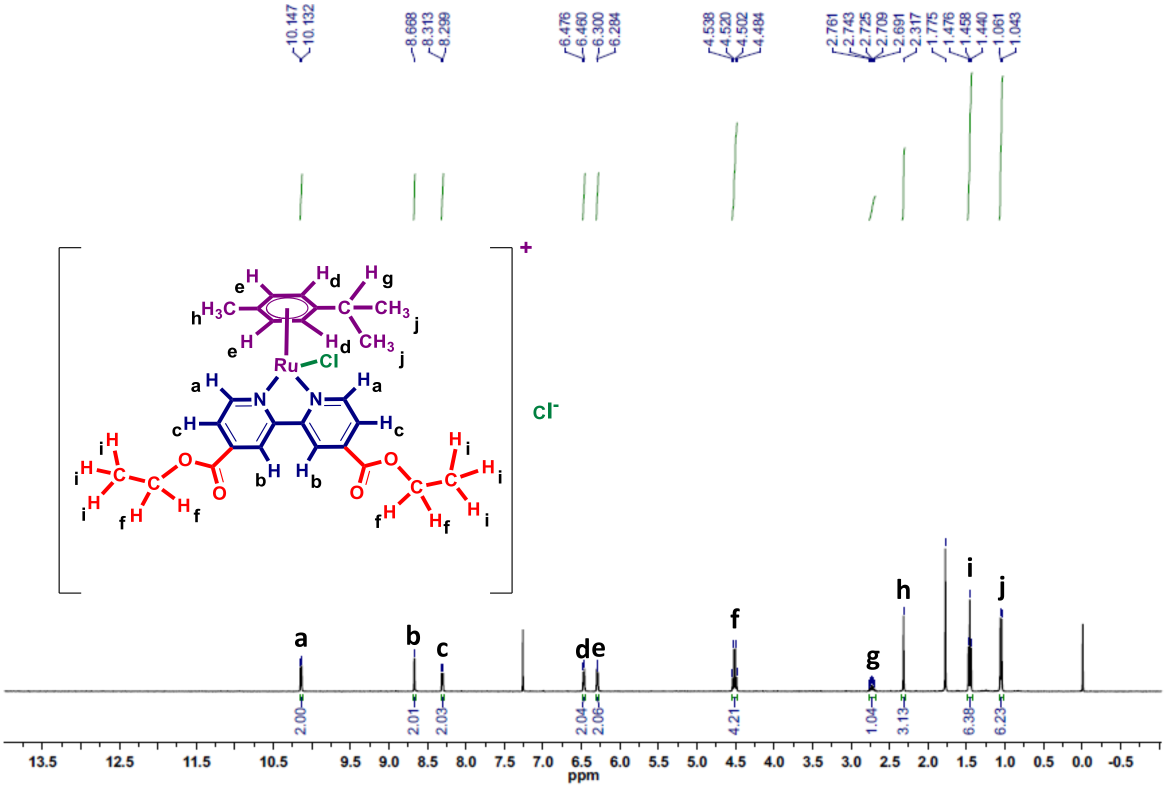

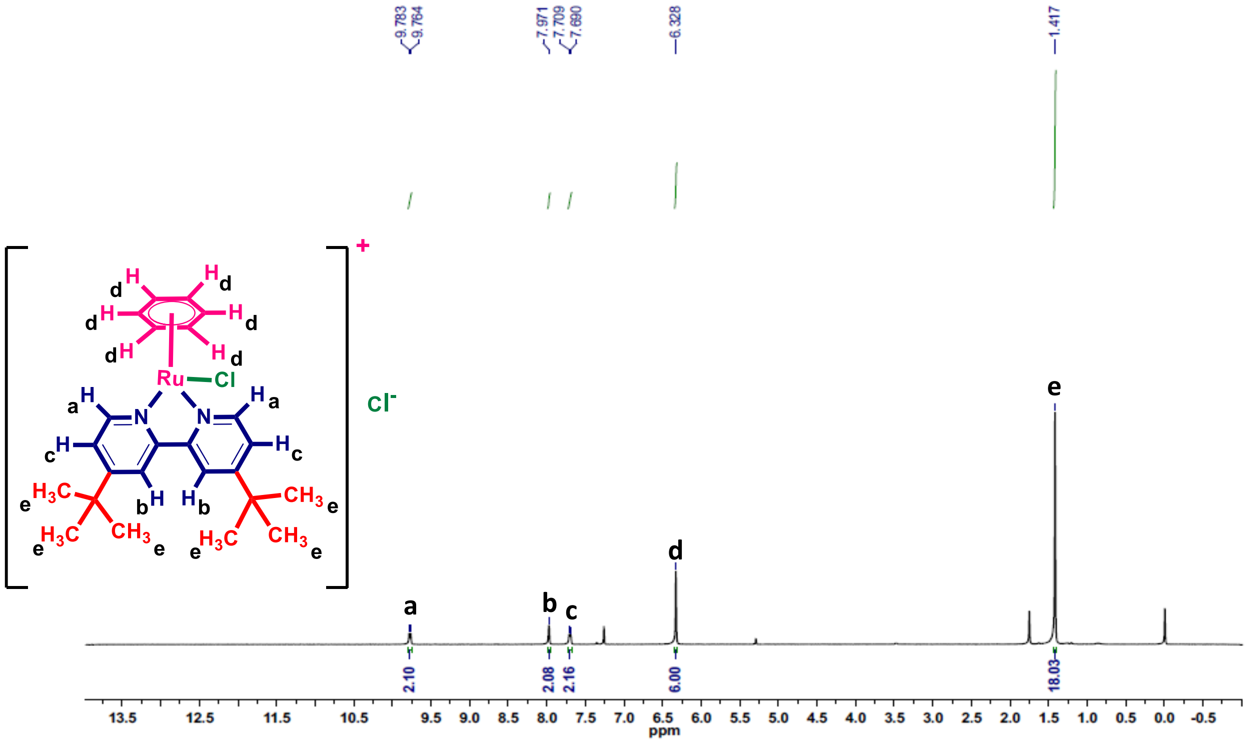
9.783
9.764
7.971
7.709
7.690
6.328
1.417
Ru
Cl
Cl⁻
a b c d e
2.10
2.08
2.16
6.00
18.03
13.5 12.5 11.5 10.5 9.5 9.0 8.5 8.0 7.5 7.0 6.5 6.0 5.5 5.0 4.5 4.0 3.5 3.0 2.5 2.0 1.5 1.0 0.5 0.0 -0.5
ppm

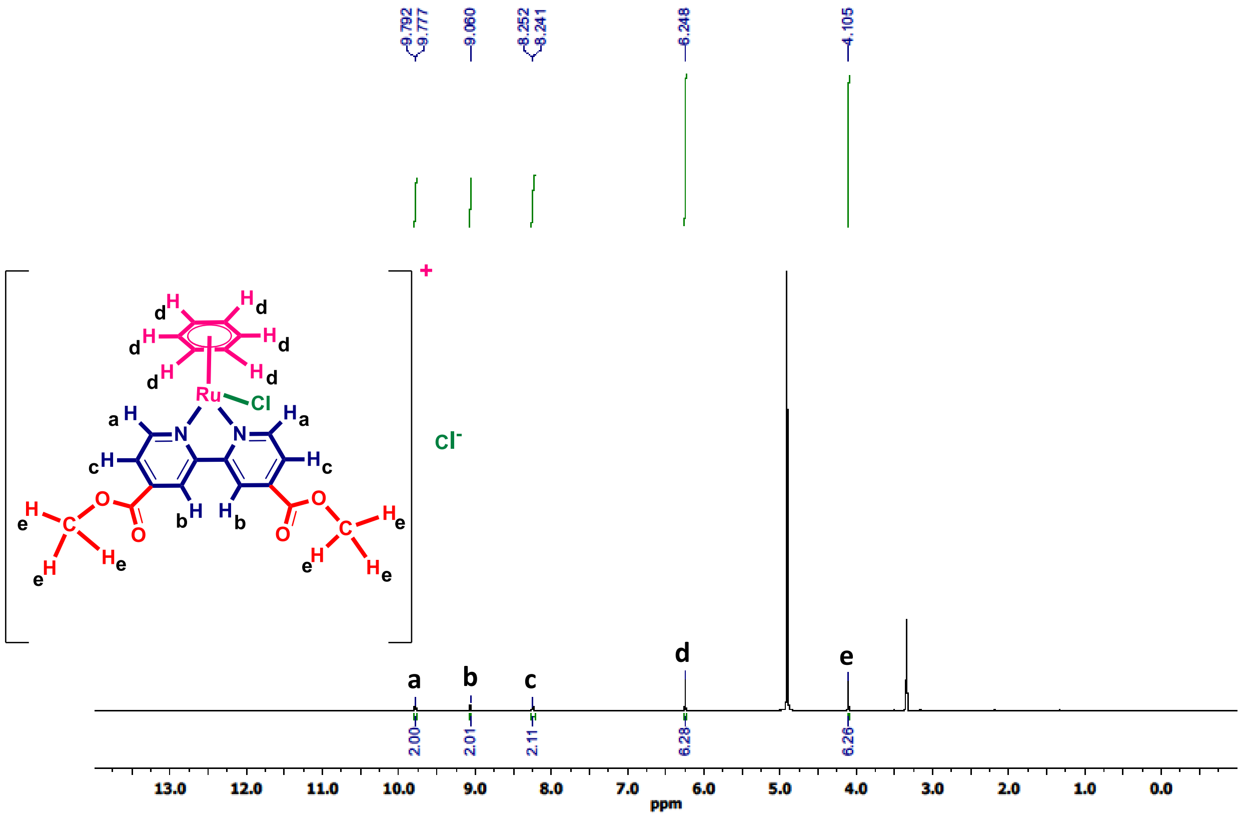
9.792
9.777
9.060
8.252
8.241
6.248
4.105
Ru
Cl
Cl⁻
a b c d e
2.00
2.01
2.11
6.28
6.26
13.0 12.0 11.0 10.0 9.0 8.0 7.0 6.0 5.0 4.0 3.0 2.0 1.0 0.0
ppm

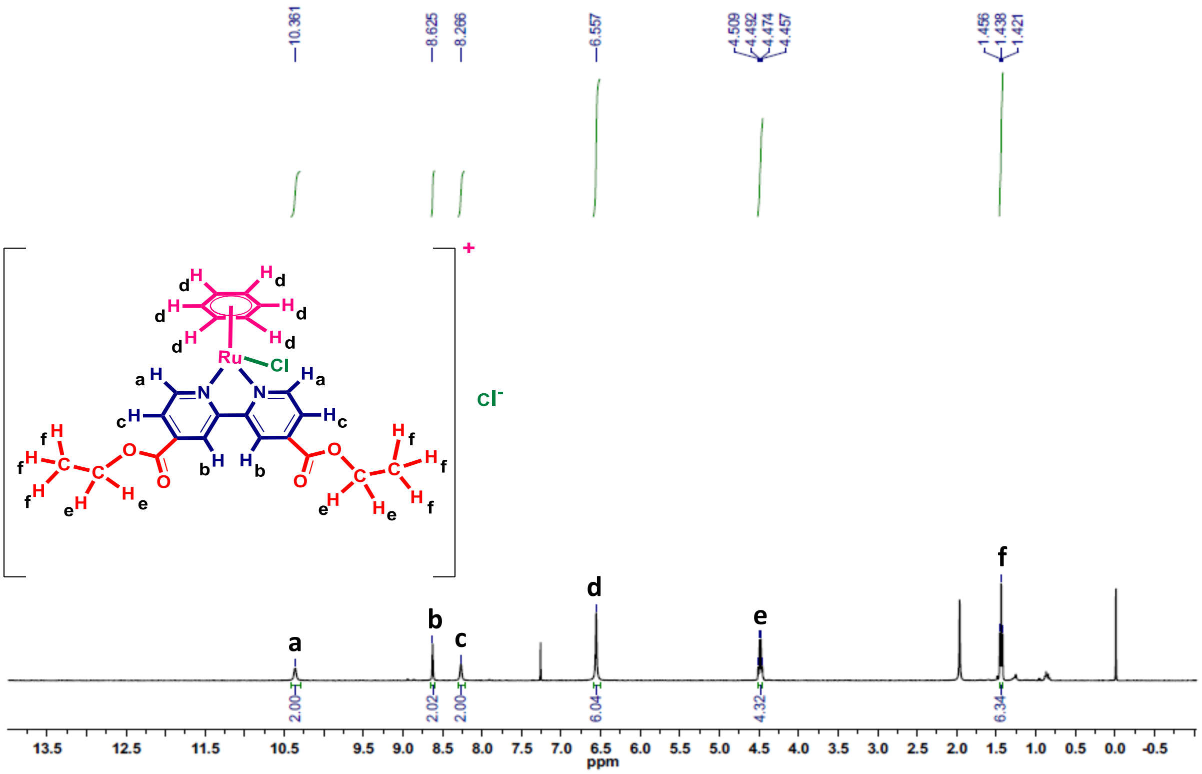

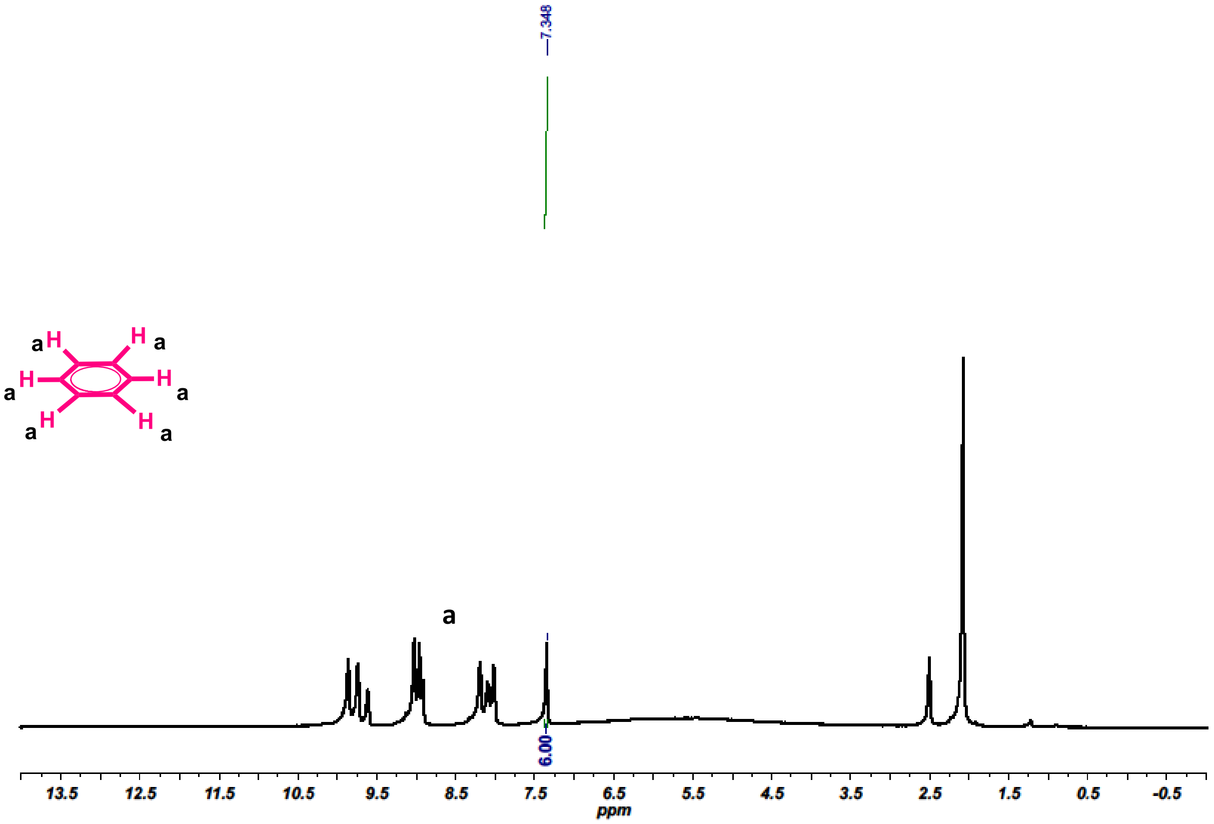

Figure S3. ^{1}H NMR spectra of complexes (**1**-**8**).

3. CT DNA binding studies

All the experiments investigating interaction mode of the complexes with CT DNA were carried out in Tris buffer solution (50 mM NaCl, 5 mM TrisHCl, pH 7.2) at room temperature. The stock CT DNA solution was prepared by diluting CT DNA in Tris buffer and the final concentration of the diluted solution (per nucleotide phosphate) was determined spectrophotometrically using the molar absorbance coefficient of 6600 M^{-1} cm^{-1} at 260 nm. Also, the above-mentioned absorbance results indicating that CT DNA was sufficiently free from protein. The observing absorption ratio of CT DNA solution was found as $\geq$ 1.8, which is used to check the purity of the stock solution. The prepared DNA stock solution were stored at 4 ^{o}C and used for within a week after their preparation. The absorption titrations of the complexes (15 µM in Tris-HCl buffer containing 5 % DMF) was titrated against CT DNA (0-50 µM). The spectra were recorded after equilibration for 3 min, allowing the compounds to bind to the CT DNA.

3.1 Absorbance titration experiments

An investigation on the binding between complexes and CT DNA were performed in spectroscopic titration at room temperature. A required concentration of the stock solution was prepared by dissolving the appropriate amount of the complexes in 5 % water (or) 5 % DMF/Tris-Hcl/Nacl. The solution was diluted in such a way that the constant concentration of the complexes 15 µM; it was treated with aliquots of a stock concentrated solution of the CT DNA in a 1 cm sample cell. Whereas a reference cell without the complexes was titrated in a same way simultaneously. The spectra were recorded after equilibration for 3 min, allowing the compounds to bind to the CT DNA and the significant absorbance change was noted. The binding constant of the complexes with CT DNA (K_b) was obtained from the ratio of slope to intercept by plotting [DNA] / (ε_a–ε_f) *versus* [DNA] according to the equation, [DNA] / (ε_a–ε_f) = [DNA] / (ε_b–ε_f) + 1 / K_b (ε_b–ε_f). Where, [DNA] is the concentration of DNA in base pairs, ε_a is the apparent extinction coefficient value found by calculating $A_{(observed)}$/[complex], ε_f is the extinction coefficient of the free compound, and ε_b is the extinction coefficient of the compound in the fully bound form. Each set of data, when fitted into the above equation, gave a straight line with a slope of 1/(ε_b–ε_f) and y-intercept of 1 / K_b (ε_b–ε_f).

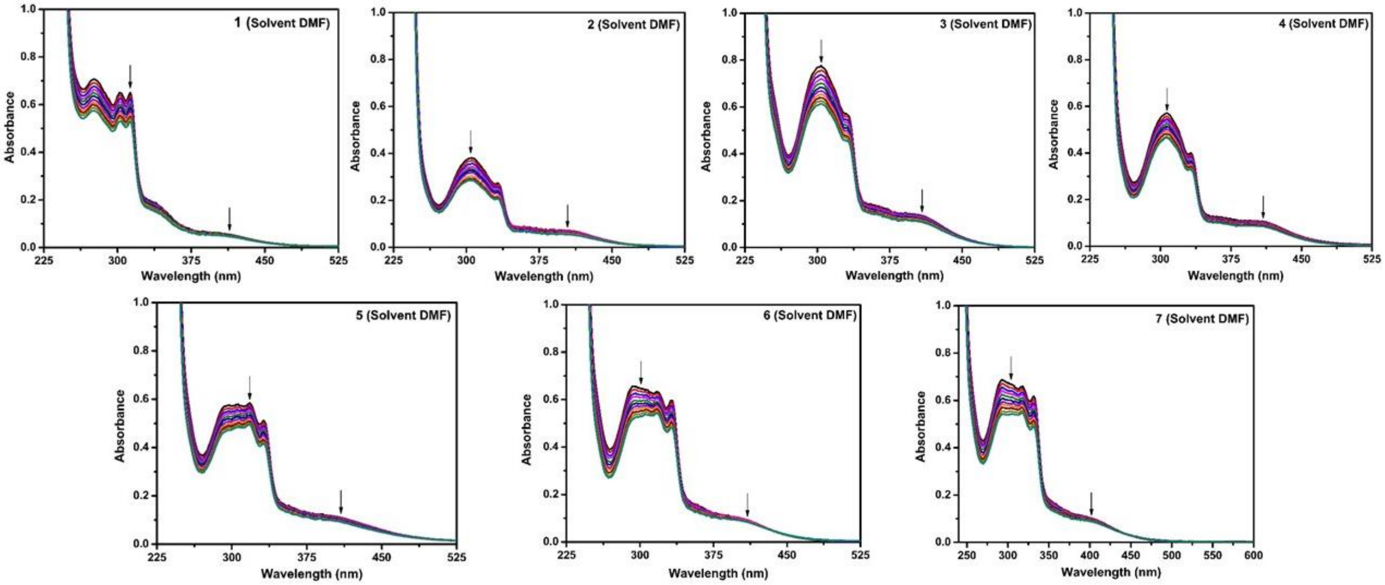

Figure S4. Absorption spectra of complexes (**1**-**7**) in Tris-HCl buffer upon addition of CT DNA. [Complex] = 1.5 × 10^{-5} M, [DNA] = 0-40 µM. Arrow shows decrease in absorption upon increasing DNA concentration.

3.2 Fluorescence titration experiments

Ethidium bromide fluorescence displacement study was investigated by fluorescence spectroscopic technique using EB-bound CT DNA solution in TrisHCl/NaCl buffer (pH 7.2). The displacement of the EB-bound was obtained by addition of the concentration solution of the complexes (0-50 µM) to the buffered solution that is 5 µM CT-DNA pre-treated with a saturating ethidium bromide 5 µM. The emission intensities were taken by exciting at 520 nm (593 nm emission wavelength) and measuring emission spectra from 530-800 nm.

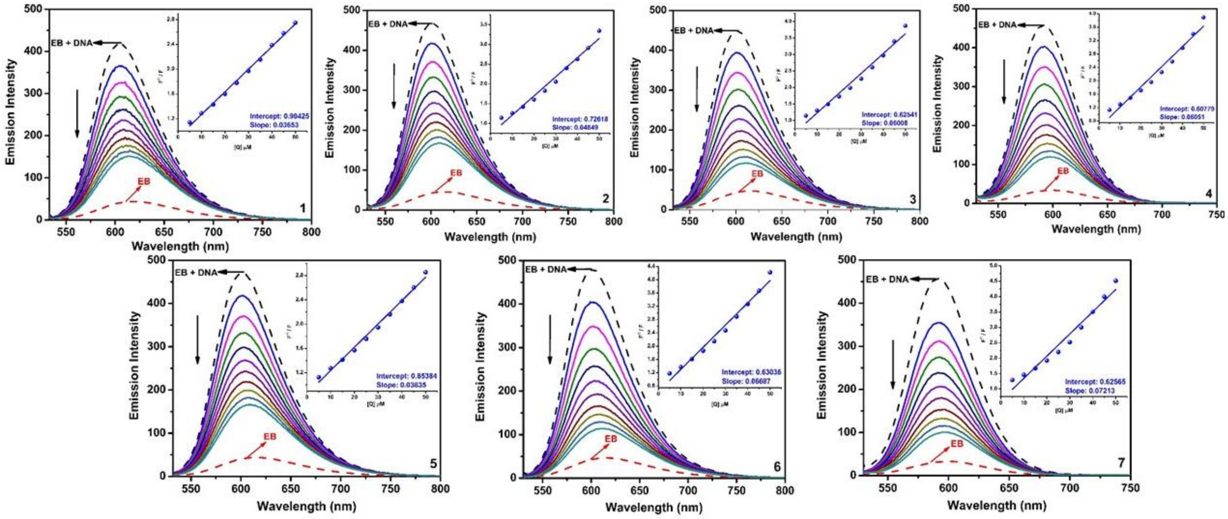

Figure S5. Fluorescence quenching curves of EB bound to DNA in the presence of complexes **1**-**7**. [DNA]= 5 µM, [EB] = 5 µM and [complex] = 0-50 µM. Arrow shows decrease in emission intensity upon increasing complexes concentration.

3.3 Viscosity measurements

Viscosity experiments were performed in Micro-Ubbelohde viscometer in an external thermostat water bath maintained at (25.0 ± 0.1 °C). Changes in the viscosity of the DNA solution (100 µM) was measured in the presence and absence of complexes concentration (0-60 µM). The flow time was measured three times for each sample and an average flow time was then calculated. The values of relative specific viscosity $(\eta/\eta^0)^{1/3}$, where η is the relative viscosity of DNA in the presence of the complex and η^0 is the relative viscosity of DNA alone, were plotted against $1/R$ ($1/R$ = [compound]/[DNA]). Relative viscosity (η^0) values were calculated from the observed flow time of the DNA solution (t) corrected for the flow time of the buffer alone (t^0), using the expression $\eta^0 = (t - t^0)/t^0$.

3.4 Binding of complexes (1-7) on CT DNA stability was assessed by agarose electrophoresis

In this experiment, 40 µM of CT DNA was treated with 100 µM of synthesized complexes (**1**-**7**), and the mixture was incubated for 4 h at 37 °C. After incubation, the reaction mixture was treated with a loading buffer in a ratio of five to one (the loading buffer contains 0.05(w/v)% of bromophenol blue and 5(v/v)% of glycerol). All testing samples were subjected to electrophoresis for 1 h at 100 eV on the 1.5% agarose gel using Tris/Borate/EDTA buffer (pH 7.3). The gel was stained with 0.5 µg mL^{-1} ethidium bromide for 10 min after electrophoresis, and then photographed on the blue light transilluminator. The extent stability of the CT DNA was determined by analysing the intensities of the bands using popular open-source software ImageJ.

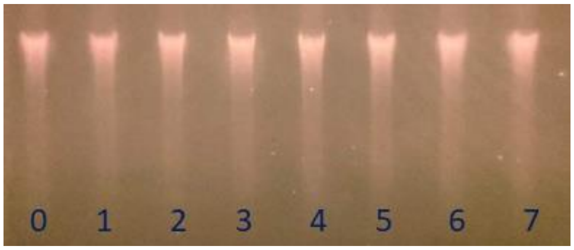

Figure S6. DNA fragmentation assay. Photograph displaying no effect of complexes (**1**-**7**) on CT DNA. Lane 0: Untreated DNA, Lane 1: DNA+**1**, Lane 2: DNA+**2**, Lane 3: DNA+**3**, Lane 4: DNA+**4**, Lane 5: DNA+**5**, Lane 6: DNA+**6** and Lane 7: DNA+**7**.

4 Interaction of complexes with Bovine serum albumin

4.1 Fluorescence titration

The interaction of complexes with BSA was studied using fluorescence spectra, recorded at a fixed excitation wavelength corresponding to BSA at 280 nm and monitoring the emission at 335 nm. Stock solution of BSA were prepared by dissolving commercial BSA in 50mM phosphate buffer (pH 7.2) and stored at 4 °C for further use. Concentrated stock solution of each test compound were prepared by dissolving it in DMF-phosphate buffer (5:95) and diluted with phosphate buffer to get required concentrations. For the fluorescence measurements, 2.5 mL solution of BSA was titrated with successive additions of 10^{-6} M stock solution of complexes (0-20 µM) using a micropipette.

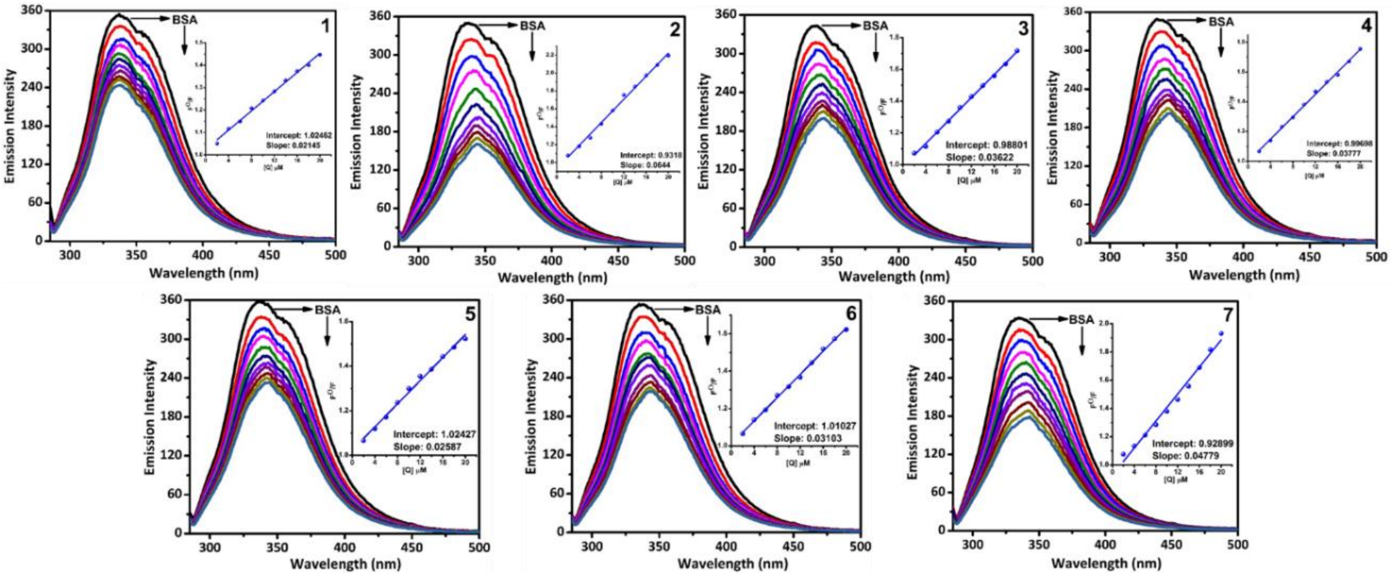

Figure S7. Fluorescence quenching curves of BSA in the absence and presence of complexes **1**-**7**. [BSA]= 1 µM and [complex] = 0-20 µM. Arrow shows decrease in emission intensity upon increasing complexes concentration.

4.2 Sample preparation of positive MALDI-TOF MS spectra

The binding of complexes (**2** or **7**) with BSA were placed into an 8 kDa molecular weight cut-off (MWCO) regenerated cellulose dialysis tubing roll. The dialysis sample was placed into a 5 L beaker containing 4 L of ultrapure water. The dialysis was performed overnight with a small (5 × 2 mm PTFE, Teflon) stir bar. After, the samples were stored at 4 °C and used for MALDI-TOF MS analysis within a week after their preparation.

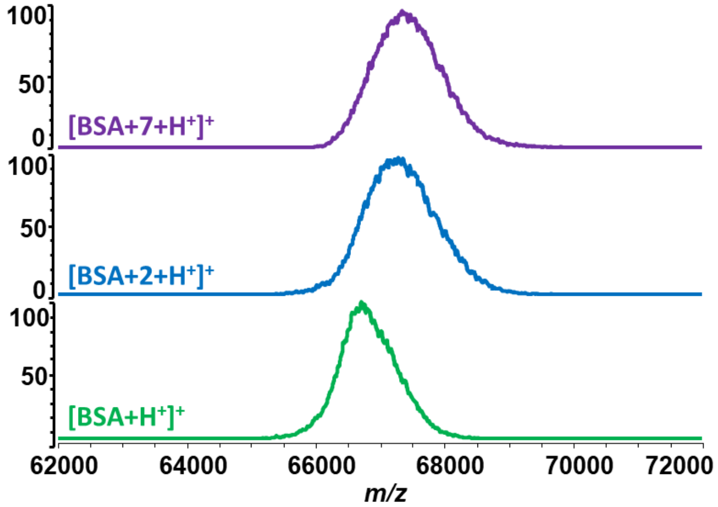

Figure S8. MALDI-MS spectra of BSA and BSA with **2** and **7**.

5. Molecular Docking Studies

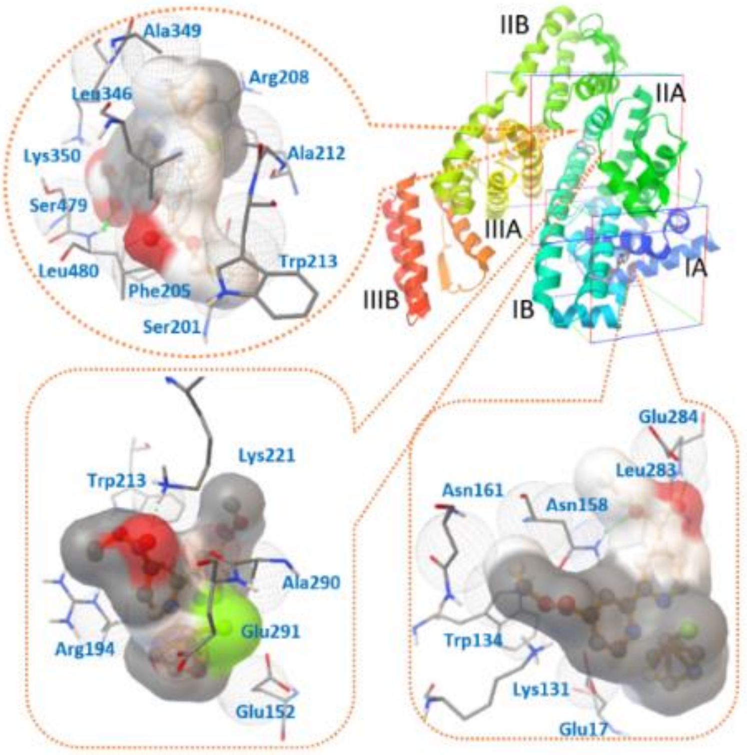

Figure S9. To accompany Figure 13, details of the interactions between **7** and residues surrounding the tryptophan residue. Mesh sphere is contact on the atom of the indicated residue that is in van der Waals contact or H-bonding with complex **7**.

6. *In vitro* cytotoxicity evaluation by MTT assay

The *In vitro* anticancer activity of the complexes (**1**-**7**) was evaluated against HepG-2, T24 and EAhy.926, and MCF-10a and Vero cells. HepG-2 and EAhy.926 cancer cells were maintained in DMEM (Dulbecco's modified Eagle's medium), T24 cancer cells in McCoy's 5a medium, and MCF-10a and Vero cells in RPMI-1640 (Roswell Park Memorial Institute medium) at 37°C under a humidified atmosphere of 5% CO_2 and 95% air. For the activity evaluation, exponentially growing cells were harvested, plated in 96-well plates (2 × 10^4 cells/well) in DMEM/ McCoy's 5a/ RPMI-1640 and kept inside the humidified incubator at 37°C for 24 h. After the cells had been washed with PBS, the medium was changed to serially diluted test samples in medium, with the control and blank in each plate. The cells were allowed to proliferate for 24 h, then washed twice with PBS, and a solution of 100 µL of medium containing 10% WST-8 (water soluble tetrazolium salts) available as cell counting kit (CCK-8) was added to each well. After incubating for 3 h, the absorbance at 570 nm was measured (Perkin Elmer EnSpire multilabel reader). Cell viability was calculated from the mean values of three wells using the following equation,

$$\textbf{Cell viability }(\%) = \frac{(\textbf{Absorbance of test sample} - \textbf{Absorbance of blank})}{(\textbf{Absorbance of control} - \textbf{Absorbance of blank})} \times \textbf{100\%}$$

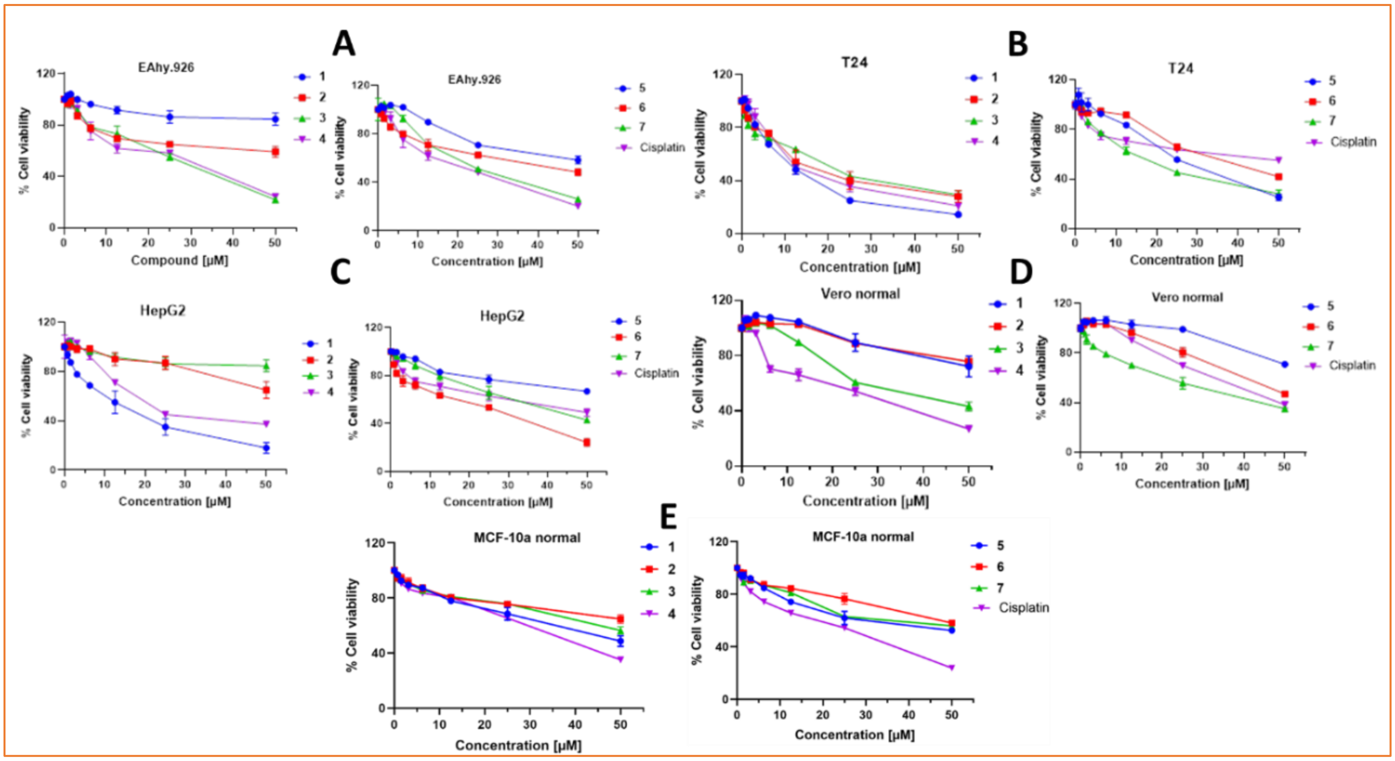

Figure S10. Cytotoxic effects of complexes (**1**-**7**) against (A) EA.hy-926, (B) HepG-2, (C) T24, (D) normal kidney (Vero) cells and (E) MCF-10a-breast epithelial. Data expressed in SD±mean with three replicates.

7. Fluorescence images

Density of 3×10^4 of urinary bladder T24 cancer cells was seeded in 30 mm plates and cells were treated with IC_{50} value of active complexes **1** and **2** for 24 h. After 24 h incubation, control and treated cells were stained with acriddine orange (AO)/ethidium bromide (EB) and incubated for 10 min. The images were taken immediately under fluorescent microscope (EVOS, 20x).

8. Flow cytometry studies

The cell apoptotic rate was determined by flow cytometry analysis with the fluorescein isothiocyanate (FITC) Annexin V apoptosis detection kit (Multi Sciences, China). T24 cancer cells were collected by trypsinization, washed twice and resuspended in 500 mL, 1 × binding buffer with 5 mL of FITC Annexin V and 10 mL of PI. After incubation for 15 min, the samples were subjected to analysis by flow cytometry. The results were analysed with the BD FACS Calibur™ system.

Table S1. Crystal data and structure refinement for **1**-**7**

⁑= Empirical formula and formula weight of the complexes were included in their corresponding solvent molecules (CH_2Cl_2 (or) H_2O).

Parameters		**1**⁑	**2**⁑	**3**⁑	**4**⁑	**5**	**6**⁑	**7**⁑
Empirical formula		$C_{29}H_{38}Cl_4N_2Ru$	$C_{44}H_{46}Cl_2N_4O_9Ru_2$	$C_{24}H_{36}Cl_2N_2O_9Ru$	$C_{28}H_{34}Cl_6N_2O_4Ru$	$C_{24}H_{30}Cl_2N_2Ru$	$C_{20}H_{22}Cl_2N_2O_6$ Ru	$C_{22}H_{28}Cl_2N_2O_7Ru$
Formula weight		678.21	1047.89	668.52	776.34	518.47	558.36	604.43
Temperature(K)		100(2)	100(2)	100(2)	100(2)	100(2)	296(2)	296
Wavelength(Å)		0.71073	0.71073	0.71073	0.71073	0.71073	0.71073	0.71073
Crystal system		Orthorhombic	Trigonal	Triclinic	Monoclinic	Triclinic	Monoclinic	Triclinic
Space group		*P2₁2₁2₁*	*P*3(2)21	*P-1*	*P2(1)/c*	*P-1*	*P2(1)/c*	*P-1*
Unit cell	*a* (Å), α (°)	16.3655(11), 90	12.7214(3), 90	6.8734(2), 95.1465(8)	15.2555(6), 90	12.345(4), 88.658(5)	6.967(13), 90	12.832(7), 75.639(11)
	b (Å), β (°)	19.1285(10), 90	12.7214(3), 90	12.1907(4), 99.6348(8)	11.0921(5), 91.6615(10)	14.726(4), 72.424(5)	16.934(3), 97.238(6)	14.051(7), 73.790(11)
	c (Å), γ (°)	42.7266(19), 90	21.9847(6), 120	17.1940(5), 95.3631(9)	19.2138(8), 90	14.951(4), 84.323(5)	19.187(4), 90	14.910(8), 84.304(12)
Volume (Å^3)		13375.5(13)	3081.21	1406.00(7)	3249.9(2)	2578.3(13)	2245.7(7)	2500(2)
Z		16	3	2	4	4	4	4
Density (Mg/m^3)		1.347	1.694	1.579	1.587	1.336	1.651	1.606
μ (mm^{-1})		0.848	0.929	0.800	1.012	0.827	0.975	0.885
F(000)		5568	1596	688	1576	1064	1128	1232
Crystal size (mm)		0.350 X 0.200 X 0.100	0.150 X 0.130 X 0.050	0.20 X 0.15 X 0.10	0.100 X 0.070 X 0.030	0.600 X 0.100 X 0.030	0.400 X 0.150 X 0.100	0.700 X 0.200 X 0.100
θ range (°)		3.2930 to 27.6040	1.848 to 24.999	2.179 to 27.498	2.120 to 26.417	1.390 to 24.999	1.610 to 24.99	1.461 to 28.323
Limiting indices		–21<=h<=10, –24<=k<=20, –55<=l<=47	–15<=h<=15, –15<=k<=15, –26<=l<=26	–8<=h<=8, –15<=h<=15, –22<=h<=22,	–18<=h<=19, –13<=k<=13, –24<=l<=16	–14<=h<=14, –17<=k<=17, –17<=l<=15	–8<=h<=8, –20<=k<=19, –22<=l<=19	–17<=h<=17, –17<=k<=17, –19<=l<=19
Reflections collected / unique		59486/30573 [R(int)]= 0.0648	37063/3607 [R(int)]= 0.0365	36284/6444 [R(int)]= 0.0365	20019/6653 [R(int)]= 0.0386	17244/8989 [R(int)]= 0.0346	9388/3942 [R(int)]= 0.0532	19379/1194 [R(int)]= 0.0315
Completeness to theta = 25.242°		99 %	100 %	99.9 %	99 %	98.8 %	99.6 %	98.3 %
Absorption correction		Semi-empirical from equivalents						
Refinement method		Full-matrix least-squares on F^2						
Goodness-of-fit on F^2		1.292	1.084	0.993	1.020	1.106	1.063	1.052
Final R indices [I>2sigma(*I*)]		R1= 0.0809, wR2= 0.1884	R1= 0.00168, wR2= 0.0412	R1= 0.0247 wR2= 0.103	R1=0.0349, wR2=0.0781	R1=0.0493, wR2=0.1440	R1=0.0500, wR2 0.1289	R1=0.0449, wR2=0.139
R indices (all data)		R1= 0.1539, wR2=0.2435	R1= 0.0173, wR2= 0.0416	R1= 0.0294 wR2= 0.108	R1=0.0483, wR2=0.0853	R1=0.0541, wR2=0.1490	R1=0.0598, wR2=0.1355	R1=0.0605, wR2=0.141
Extinction cofficient		n/a						
max/min (e.Å^{-3})		1.838 & –2.136	1.333 & –0.252	0.745 & –0.994	1.645 & –0.920	3.307 & –1.449	1.257 & –0.584	0.801 & –0.639

Table S2. Selected bond lengths (Å), angles (°) of **1**-**7**

Bond lengths (Å)	**1**	**2**	**3**	**4**	**5**	**6**	**7**
Ru(1)–Cl(1)	2.423(4)	2.406(8)	2.406(4)	2.387(7)	2.39(11)	2.38(15)	2.40(13)
Ru(1)–N(1)	2.097(11)	2.079(3)	2.072(15)	2.067(2)	2.088(3)	2.075(4)	2.092(3)
Ru(1)–N(2)	2.115(10)	2.082(2)	2.1653(17)	2.083(2)	2.091(3)	2.077(4)	2.088(3)
Ru(1)–C(20)/C(1)/C(16)/C(1)/C(1)/C(1)/C(1)	2.228(14)	2.444(3)	2.2175(18)	2.201(3)	2.183(5)	2.169(7)	2.178(4)
Ru(1)–C(21)/C(2)/C(17)/C(2)/C(2)/C(2)/C(2)	2.248(13)	2.211(3)	2.1653(17)	2.161(3)	2.204(5)	2.168(6)	2.197(4)

Ru(1)–C(22)/C(3)/C(18)/C(3)/C(3)/C(3)/C(3)	2.184(14)	2.164(3)	2.2218(17)	2.219(3)	2.181(5)	2.166(7)	2.202(4)
Ru(1)–C(23)/C(4)/C(19)/C(4)/C(4)/C(4)/C(4)	2.198(12)	2.208(3)	2.2471(17)	2.241(3)	2.188(5)	2.191(7)	2.213(4)
Ru(1)–C(24)/C(5)/C(20)/C(5)/C(5)/C(5)/C(5)	2.205(11)	2.178(3)	2.2070(17)	2.201(3)	2.187(5)	2.183(6)	2.195(4)
Ru(1)–C(25)/C(6)/C(21)/C(6)/C(6)/C(6)/C(6)	2.220(13)	2.201(3)	2.2034(17)	2.184(3)	2.179(5)	2.193(6)	2.213(4)
N(1)–C(5)/C(11)/C(5)/C(15)/C(11)/C(11)/C(11)	1.365(15)	1.358(4)	1.357(2)	1.360(4)	1.355(5)	1.359(6)	1.354(4)
N(2)–C(6)/C(16)/C(6)/C(16)/C(12)/C(12)/C(12)	1.364(15)	1.360(4)	1.360(2)	1.355(4)	1.360(5)	1.362(6)	1.364(4)
O(1)–⁑/C(21)/C(11)/C(22)/⁑/C(17)/C(17)	-	1.215(4)	1.205(2)	1.450(4)	-	1.176(6)	1.195(5)
O(2)–⁑/C(22)/C(12)/C(21)/⁑/C(18)/C(18)	-	1.223(4)	1.452(2)	1.202(4)	-	1.303(7)	1.467(4)
Bond angles (°)							
N(1)–Ru(1)–Cl(1)	85.5(3)	89.83(8)	83.10(4)	85.7(7)	84.01(9)	85.63(12)	85.32(9)
N(2)–Ru(1)–Cl(1)	83.7(3)	83.11(8)	84.66(4)	85.2(7)	83.43(9)	84.76(11)	85.22(8)
N(1)–Ru(1)–N(2)	76.7(4)	76.3(10)	77.06(6)	76.8(9)	76.6(13)	76.99(15)	76.8(11)
C(20)/C(1)/C(16)/C(1)/C(1)/C(1)/C(1)–Ru(1)–Cl(1)	92.1(4)	87.77(9)	163.76(5)	167.91(8)	87.47(14)	133.5(2)	134.3(15)
C(21)/C(2)/C(17)/C(2)/C(2)/C(2)/C(2)–Ru(1)–Cl(1)	93.0(4)	106.9(9)	149.58(5)	143.63(8)	104.7(16)	101.2(2)	101.3(14)
C(22)/C(3)/C(18)/C(3)/C(3)/C(3)/C(3)–Ru(1)–Cl(1)	120.1(4)	143.1(9)	112.88(5)	108.11(8)	139.4(16)	88.4(4)	88.57(13)
C(23)/C(4)/C(19)/C(4)/C(4)/C(4)/C(4)–Ru(1)–Cl(1)	158.3(4)	165.5(8)	91.21(5)	89.70(8)	165.8(14)	106.2(2)	105.4(16)
C(24)/C(5)/C(20)/C(5)/C(5)/C(5)/C(5)–Ru(1)–Cl(1)	153.9(4)	128.7(9)	97.06(5)	99.46(8)	133.0(16)	140.1(2)	139.3(16)
C(25)/C(6)/C(21)/C(6)/C(6)/C(6)/C(6)–Ru(1)–Cl(1)	117.3(5)	97.10(9)	125.9(5)	130.8(8)	99.91(16)	166.7(18)	167.9(14)
C(5)/C(11)/C(5)/C(15)/C(11)/C(11)/C(11)–N(1)–Ru(1)	117.8(9)	118.2(2)	117.2(11)	117.6(19)	115.5(3)	117.5(3)	117.2(2)
C(1)/C(16)/C(1)/C(16)/C(12)/C(12)/C(12)–N(1)–Ru(1)	124.9(9)	117.5(19)	123.6(12)	116.9(18)	116.1(3)	117.2(3)	117.2(2)

⁑=Absence of carbon-oxygen bonds

Optimized Cartesian coordinates for complex 1-7 in H2O at the ωB97XD level of theory with the Stuttgart-Dresden relativistic effective core potential and its associated basis sets SDD for Ru and the 6-31+G(d,p) for all other atoms.

Complex 1:

Ru	1.80568500	-0.25760200	-0.39930600
Cl	1.44180700	-0.64024800	-2.79390300
N	0.23666800	1.13975000	-0.53314300
N	0.08643300	-1.42631900	-0.11573900
C	0.39960500	2.44422000	-0.77911700
H	1.40734600	2.77464900	-0.99783300
C	-0.65320600	3.34667500	-0.75879000
H	-0.43477500	4.38802000	-0.95824100
C	-1.94718600	2.90324100	-0.48284800
C	-2.10323200	1.53154000	-0.25655900
H	-3.08573200	1.12478800	-0.05017000
C	-1.01043400	0.67963400	-0.28788800
C	-1.09821100	-0.77707300	-0.07403700
C	-2.28865200	-1.45746100	0.12740100
H	-3.21805500	-0.90236500	0.15224500
C	-2.30065300	-2.84708900	0.28964100
C	-1.06580200	-3.49323700	0.21876200
H	-0.97186800	-4.56690000	0.32026500
C	0.09178500	-2.75787400	0.01164300
H	1.05372900	-3.24940700	-0.06213400
C	-3.16170500	3.82727000	-0.42601600
C	-2.78514800	5.28816200	-0.69708400
H	-2.07463900	5.66802200	0.04482400
H	-2.34936200	5.41769000	-1.69346200
H	-3.68681800	5.90623400	-0.64416100
C	-3.79414700	3.72942800	0.97548400

H	-3.07842500	4.02914800	1.74834600
H	-4.66029200	4.39718900	1.03280900
H	-4.13689000	2.71421800	1.19781900
C	-4.18214600	3.37300900	-1.48665600
H	-4.52689900	2.34982000	-1.30835200
H	-5.05602300	4.03258700	-1.45971800
H	-3.74758000	3.42074800	-2.49078500
C	-3.62358800	-3.57386400	0.52314200
C	-3.42999700	-5.08741300	0.66754700
H	-2.99383600	-5.52893900	-0.23478000
H	-2.78969200	-5.33603500	1.52059000
H	-4.40392600	-5.55878400	0.83218300
C	-4.26419200	-3.02916300	1.81408800
H	-4.47105800	-1.95695500	1.74170100
H	-5.21322400	-3.54373200	1.99787300
H	-3.60980200	-3.19691300	2.67611400
C	-4.55945400	-3.30543000	-0.67068300
H	-4.77070600	-2.23861200	-0.79219600
H	-4.11973100	-3.67765200	-1.60209100
H	-5.51246100	-3.82079500	-0.51145400
C	4.41775600	-2.16815800	-1.42834300
H	3.94164500	-3.14433000	-1.31166200
H	4.27448100	-1.82218800	-2.45443000
H	5.49244700	-2.28235200	-1.25151400
C	3.85507400	-1.17971600	-0.45396900
C	3.95044700	0.23241000	-0.70712100
H	4.39506000	0.57612300	-1.63436700
C	3.41013600	1.16597400	0.18498500
H	3.45721100	2.22273600	-0.05623200
C	2.68669800	0.74467100	1.35260800
C	2.59067300	-0.64281700	1.60036200
H	2.00996500	-1.00923500	2.43861100
C	3.18932400	-1.58993200	0.71267400
H	3.05072600	-2.64921600	0.89973300
C	2.07385800	1.78712800	2.26192400
H	1.80649900	2.64006000	1.62726100
C	3.14302100	2.25632600	3.25837400
H	4.03077700	2.63632700	2.74278000
H	2.74318700	3.05776000	3.88718900
H	3.44998400	1.43029400	3.90976200
C	0.81128300	1.31622500	2.98271700
H	0.06772400	0.92515600	2.28115600
H	1.03382300	0.53804300	3.72028900
H	0.36318400	2.15936100	3.51656600

Complex 2:

Ru	-1.51149200	0.14412900	-0.41361600
Cl	-1.16057300	0.48222800	-2.80832900
O	4.74731200	3.07262800	0.66763300
H	5.48064000	3.68039200	0.85730300
O	4.70502600	-3.30769800	-0.92042800
H	4.66298100	-2.43034400	-1.32880100
N	0.15128700	1.39881900	-0.15268200
N	0.11961700	-1.18040800	-0.51531000
O	3.54888600	4.96843200	0.77336900
O	3.42885600	-4.88550000	-0.06092500
C	2.48260300	-1.45122400	-0.28661800
H	3.45277800	-1.01076300	-0.08971900
C	-0.00184500	-2.49281100	-0.75697500
H	-0.99772400	-2.86176600	-0.96357900
C	1.34167500	-0.65598200	-0.28943400
C	0.06051600	2.72959700	-0.02960200

H	-0.92526700	3.16392000	-0.13142300
C	1.35955000	0.80241200	-0.07592700
C	1.08696300	-3.34843300	-0.74891000
H	0.94284400	-4.40573400	-0.93519200
C	-2.59274600	-2.08674600	3.50799000
H	-3.01878400	-1.24805700	4.06991900
H	-2.10435500	-2.76264600	4.21653300
H	-3.41264500	-2.62909400	3.02620100
C	-3.64924500	0.83271000	-0.56246300
C	1.16908900	3.52270100	0.21557800
H	1.05257900	4.59426500	0.32107200
C	3.61237000	3.76360400	0.61000700
C	-2.29899300	-0.72216100	1.46271800
C	-3.03673300	1.45324700	0.53825800
H	-3.01490900	2.53597300	0.59579900
C	-1.57480900	-1.57976600	2.47706700
H	-1.18375200	-2.44653900	1.93060400
C	2.35149200	-2.81652500	-0.51464200
C	-3.59205800	-0.60142600	-0.64516400
H	-3.99662000	-1.10146900	-1.51764600
C	-2.95933500	-1.35070500	0.35442200
H	-2.88581400	-2.42745800	0.24203500
C	-2.34416700	0.68785300	1.52766800
H	-1.80085700	1.21523800	2.30322700
C	2.51565300	1.53591800	0.16712100
H	3.47814900	1.04704500	0.23546200
C	2.41474700	2.91369400	0.32552900
C	3.52663100	-3.74884500	-0.48115300
C	-4.31248000	1.62930400	-1.64242300
H	-5.39026900	1.66251900	-1.45078600
H	-4.15216200	1.16666500	-2.61841800
H	-3.93195800	2.65264900	-1.66407900
C	-0.40162800	-0.87281300	3.15357900
H	0.29784100	-0.45928700	2.41997600
H	0.14345200	-1.58801700	3.77634100
H	-0.74121400	-0.06042700	3.80486400

Complex 3:

Ru	-1.53129200	-0.60194700	0.04040900
Cl	-1.45113300	-0.29697600	-2.38720000
O	4.57323900	-4.17702200	-0.53122600
O	5.28597300	-2.08381000	-0.12727000
O	3.75133600	4.07536000	0.18438100
O	1.99265000	5.47476600	0.21000800
N	0.37903100	-1.44422200	-0.17509200
N	-0.26235500	1.06768000	0.10219800
C	0.60028800	-2.75093100	-0.36976300
H	-0.27169200	-3.38232800	-0.47818800
C	1.87838900	-3.28119300	-0.43215800
H	2.01459200	-4.34481400	-0.58410000
C	2.96457500	-2.42411100	-0.28984700
C	2.73787700	-1.06258100	-0.11898700
H	3.56885300	-0.37666400	-0.02155600
C	1.42771000	-0.59944000	-0.07648400
C	1.06486200	0.82317900	0.04937000
C	1.99127100	1.85801600	0.07603200
H	3.05433100	1.65886600	0.02897900
C	1.53693600	3.16957000	0.15218400
C	0.16729800	3.41337800	0.18777000
H	-0.23624900	4.41652400	0.23738300
C	-0.69954100	2.33233000	0.15680700
H	-1.77135100	2.47995900	0.17131000

C	4.34823100	-2.99570800	-0.33253900
C	6.65119800	-2.54261100	-0.14255200
H	7.25215000	-1.65530700	0.04516200
H	6.80276900	-3.28332800	0.64469300
H	6.88834900	-2.97007000	-1.11826000
C	2.54873500	4.27353800	0.18366400
C	2.88816900	6.60197900	0.24100300
H	2.24369100	7.47870800	0.24611900
H	3.49757000	6.56829300	1.14590000
H	3.52355500	6.59766600	-0.64646000
C	-0.92979000	-2.35745000	2.82100700
H	-0.36366200	-3.12341300	2.28480800
H	-0.23515700	-1.62612800	3.24015000
H	-1.45959000	-2.84466000	3.64612700
C	-1.92410200	-1.69256600	1.91874200
C	-2.26872600	-0.33239400	2.07745600
H	-1.77831300	0.25707300	2.84437400
C	-3.19977900	0.30106400	1.19659000
H	-3.39518400	1.35883200	1.32675600
C	-3.76469900	-0.39248500	0.11349300
C	-3.41198800	-1.77815600	-0.04643300
H	-3.78096100	-2.31915100	-0.91092800
C	-2.53868400	-2.41562300	0.84255800
H	-2.25228200	-3.44630500	0.66338500
C	-4.71190600	0.24532800	-0.87779000
H	-4.54943600	-0.26675700	-1.83253900
C	-6.15218300	-0.02853700	-0.42257400
H	-6.85947500	0.35663600	-1.16360300
H	-6.33525100	-1.10091900	-0.29963000
H	-6.35315500	0.46621800	0.53440600
C	-4.46518900	1.73694200	-1.09239700
H	-5.11940600	2.10275100	-1.88946200
H	-4.68638200	2.31963400	-0.19165900
H	-3.42825400	1.92516700	-1.38657000

Complex 4:

Ru	-2.03637300	-0.43245700	0.05261800
Cl	-1.75773100	-0.10205500	-2.35478500
N	-0.22181800	-1.48344000	-0.08655600
N	-0.59117400	1.08282100	0.22203100
C	-2.70766100	-1.54969400	1.84016500
C	-2.87939200	-0.16498000	2.05545700
H	-2.37830900	0.31340700	2.88969600
C	-3.64961900	0.63006100	1.15156800
H	-3.71762400	1.69669300	1.33023800
C	-4.21784800	0.07589000	-0.00728500
C	-4.03946800	-1.33434200	-0.22338000
H	-4.41023400	-1.77814600	-1.14078600
C	-3.32869700	-2.12895400	0.68384200
H	-3.17290900	-3.18002500	0.46646300
C	-1.88697900	-2.38801500	2.77186900
H	-1.13022400	-1.78305700	3.27593800
H	-1.39721900	-3.20773300	2.24079700
H	-2.54386100	-2.82422700	3.53170100
C	-4.99914900	0.88958200	-1.01459000
H	-4.87245200	0.38892700	-1.98059900
C	-4.50372600	2.32745400	-1.15732200
H	-4.66540400	2.90543900	-0.24110700
H	-5.05360100	2.82439100	-1.96228200
H	-3.43801600	2.35256200	-1.40428500
C	-6.48578200	0.84075300	-0.63523500
H	-6.84809800	-0.19018900	-0.56718800

H	-7.08222000	1.36432200	-1.38879600
H	-6.65137300	1.32743700	0.33263200
C	-0.13575100	-2.80707000	-0.27773200
H	-1.06841600	-3.34678400	-0.37204400
C	1.08069600	-3.46402000	-0.36140800
H	1.10569600	-4.53619600	-0.51320500
C	2.25076200	-2.72071300	-0.24848600
C	2.16457200	-1.34450000	-0.07150100
H	3.06357100	-0.74858600	0.00986900
C	0.90966700	-0.75141500	-0.00161700
C	0.70101500	0.69881000	0.14623200
C	1.73646000	1.62565300	0.17444000
H	2.76784700	1.30773400	0.09994300
C	1.42861300	2.97646100	0.28991900
C	0.09505100	3.36378300	0.36613400
H	-0.18606200	4.40634700	0.45160700
C	-0.88549300	2.38656400	0.31935500
H	-1.93349100	2.65049000	0.35445200
C	3.57205200	-3.42250100	-0.33072800
C	5.92751300	-3.17417200	-0.24487200
H	6.04611700	-3.65159900	-1.22057400
H	6.00383000	-3.93349700	0.53718400
C	6.91356100	-2.04993400	-0.04141400
H	6.81328200	-1.29194100	-0.82340400
H	7.92703400	-2.45851000	-0.08607500
H	6.77498100	-1.57654200	0.93464100
C	2.49761700	4.02592400	0.32610300
C	4.82169900	4.45472300	0.12390100
H	4.86577200	4.93005200	1.10674200
H	4.63094500	5.21702500	-0.63528500
C	6.06632500	3.65584800	-0.17744300
H	6.23592400	2.88964400	0.58433500
H	6.92641600	4.33133900	-0.18498300
H	5.99564000	3.17592500	-1.15751000
O	4.59619500	-2.59953100	-0.18436900
O	3.67356100	-4.62430300	-0.51181800
O	3.70487200	3.52788700	0.12182500
O	2.26150200	5.20663400	0.51984500

Complex 5:

Ru	-2.14994800	-0.00079400	0.02246800
Cl	-1.81297100	0.00107500	2.44193100
N	-0.50169400	1.29865300	-0.01859400
N	-0.50077900	-1.29923000	-0.01768800
C	-4.33658300	-0.00332400	0.36874200
H	-4.81146100	-0.00455200	1.34254500
C	-3.98748900	1.23193500	-0.23072500
H	-4.21415400	2.16736800	0.26498700
C	-3.23935000	1.22035200	-1.43573700
H	-2.87810600	2.15600600	-1.84851700
C	-2.87802000	0.00006100	-2.06289900
H	-2.27036000	0.00136300	-2.95905000
C	-3.23670800	-1.22182900	-1.43760900
H	-2.87346200	-2.15612100	-1.85171500
C	-3.98510200	-1.23679500	-0.23259700
H	-4.21013800	-2.17347000	0.26150200
C	-0.59921700	2.63046000	0.05654300
H	-1.59837600	3.04141200	0.13744500
C	0.51155800	3.46003100	0.04309500
H	0.34803100	4.52797800	0.10974900
C	1.79166200	2.91162600	-0.05075900
C	1.87327300	1.51578300	-0.11139700

H	2.84129800	1.03392800	-0.17232800
C	0.72615000	0.73783200	-0.08520000
C	0.72667000	-0.73756500	-0.08490200
C	1.87435300	-1.51467700	-0.11130000
H	2.84201200	-1.03214000	-0.17271700
C	1.79379800	-2.91056300	-0.05037200
C	0.51411700	-3.45987200	0.04399000
H	0.35138500	-4.52793200	0.11081600
C	-0.59726600	-2.63111200	0.05764800
H	-1.59610500	-3.04279500	0.13880600
C	3.06709400	3.75215700	-0.07686200
C	3.81880200	3.47267900	-1.39193300
H	4.09257500	2.41746700	-1.48765600
H	4.74039000	4.06365200	-1.41949200
H	3.20782400	3.74943500	-2.25786900
C	3.95167100	3.35186700	1.11928300
H	3.43028300	3.52312600	2.06698900
H	4.86551000	3.95547500	1.11665600
H	4.24522600	2.29860600	1.07309300
C	2.76917900	5.25338200	0.01152300
H	2.15584200	5.59632900	-0.82852100
H	3.71315400	5.80641800	-0.01593300
H	2.25730800	5.51129500	0.94473800
C	3.06984300	-3.75015900	-0.07669900
C	3.82118400	-3.47020700	-1.39188400
H	4.09430400	-2.41483200	-1.48766900
H	3.21026400	-3.74734600	-2.25773900
H	4.74313600	-4.06060200	-1.41956300
C	2.77301600	-5.25158500	0.01178800
H	3.71737500	-5.80395400	-0.01587000
H	2.15973600	-5.59497000	-0.82812200
H	2.26153600	-5.50984800	0.94511800
C	3.95430900	-3.34915900	1.11929900
H	3.43322400	-3.52084600	2.06709400
H	4.24695700	-2.29564400	1.07306600
H	4.86865200	-3.95200000	1.11650100

Complex 6:

Ru	-2.14580300	-0.22972200	0.01184800
Cl	-1.83418800	-0.21878800	2.42809100
N	-0.69704200	1.29227900	0.00493100
N	-0.32717700	-1.28453600	-0.02120900
C	-3.38784100	0.84589600	-1.43841600
H	-3.16617900	1.83131500	-1.83386000
C	-4.13791800	0.72779700	-0.24043600
H	-4.50133600	1.61201300	0.26754700
C	-4.31287000	-0.55515300	0.33401500
H	-4.79003900	-0.64358400	1.30270500
C	-3.77872900	-1.71184400	-0.28355500
H	-3.86995700	-2.67992500	0.19228900
C	-3.03305700	-1.56837400	-1.48218900
H	-2.53772700	-2.43413500	-1.90853200
C	-2.84951500	-0.29719500	-2.08222800
H	-2.24152400	-0.19182000	-2.97181200
C	-0.99907200	2.59533300	0.08417400
H	-2.04883200	2.84929900	0.15903200
C	-0.02275200	3.57767800	0.07789300
H	-0.30597400	4.62120800	0.14123000
C	1.31128100	3.19302500	-0.01048400
C	1.62672300	1.83997000	-0.07132100
H	2.65956700	1.52236400	-0.12617400
C	0.59547200	0.90787700	-0.05381400

C	0.80512900	-0.55089700	-0.06636600
C	2.05491200	-1.15759800	-0.09169600
H	2.96328300	-0.57019000	-0.13346800
C	2.13324800	-2.54483300	-0.05324000
C	0.96107000	-3.29158500	0.02196600
H	0.97162000	-4.37298000	0.06809300
C	-0.25099200	-2.62034300	0.03797800
H	-1.18693700	-3.16012300	0.10450500
C	2.37217000	4.25051200	-0.02555500
C	4.67671100	4.69414900	-0.21928500
H	4.53096800	5.42003300	-1.02109500
H	5.56801100	4.09726500	-0.40118600
H	4.74470600	5.20062200	0.74510000
C	3.48829000	-3.18264800	-0.09186700
C	4.69065900	-5.20434500	-0.00531400
H	5.18342400	-5.04005600	-0.96523100
H	4.43428200	-6.25436900	0.11902000
H	5.32875900	-4.86608900	0.81298000
O	2.12969300	5.43710900	0.10972400
O	3.58669700	3.75342100	-0.20184100
O	4.52021600	-2.54694400	-0.21950300
O	3.43495000	-4.50013900	0.02691700

Complex 7:

Ru	2.41370800	-0.55028700	0.05909600
Cl	1.93702400	-0.51407500	2.44884200
N	1.18577800	1.15476900	-0.01418800
N	0.47772500	-1.35258600	-0.11423700
C	3.90558200	0.36831400	-1.26878200
H	3.85432100	1.38145000	-1.65291100
C	4.53259900	0.12611800	-0.02080100
H	4.97467600	0.94153900	0.53735000
C	4.48066900	-1.17934900	0.52862300
H	4.86437800	-1.34985600	1.52753700
C	3.84527600	-2.23946000	-0.16120100
H	3.76268600	-3.21863100	0.29309900
C	3.22016200	-1.97079700	-1.40675000
H	2.64322100	-2.75067900	-1.89201400
C	3.26253500	-0.67587200	-1.98203400
H	2.74623700	-0.46999600	-2.91117800
C	1.65340000	2.40508000	0.09763800
H	2.71983100	2.51833900	0.24568100
C	0.82023400	3.50935700	0.03374800
H	1.23753900	4.50479300	0.12304700
C	-0.54416400	3.30789600	-0.14736900
C	-1.03414900	2.00922600	-0.23496400
H	-2.09422300	1.83320900	-0.36025000
C	-0.14014500	0.94708400	-0.15707100
C	-0.54171600	-0.47068500	-0.19436300
C	-1.86102100	-0.89956800	-0.26813200
H	-2.67610600	-0.18952300	-0.32599700
C	-2.13059600	-2.26293400	-0.25652800
C	-1.07441200	-3.16420700	-0.16100800
H	-1.23540700	-4.23424600	-0.13550500
C	0.21669700	-2.66589700	-0.08596900
H	1.06529000	-3.33192500	0.00516700
C	-3.56071900	-2.70771600	-0.33136100
C	-5.01532900	-4.59059000	-0.41676400
H	-4.86804900	-5.56684800	-0.87830800
H	-5.63389500	-3.97137200	-1.06875200
C	-5.59571600	-4.71297900	0.97554100
H	-4.95388900	-5.32952400	1.61098200

H	-6.57620400	-5.19343700	0.90634800
H	-5.72500100	-3.73262200	1.44183400
C	-1.44746000	4.50159800	-0.23484400
C	-3.69837400	5.23248300	-0.50292800
H	-3.27937300	6.04617600	-1.09773200
H	-4.52551600	4.77635000	-1.04644800
C	-4.12297100	5.68878000	0.87622900
H	-4.52735700	4.85269000	1.45332400
H	-4.90586800	6.44585700	0.77096000
H	-3.28804100	6.13222100	1.42491200
O	-4.49456600	-1.92449200	-0.35603600
O	-3.67825200	-4.02482600	-0.37218200
O	-1.03498400	5.64568000	-0.15244000
O	-2.71452700	4.16705900	-0.41786500
